# Embryonic Arsenic Exposure Triggers Long-Term Behavioral Impairment with Metabolite Alterations in Zebrafish

**DOI:** 10.3390/toxics10090493

**Published:** 2022-08-24

**Authors:** Noraini Abu Bakar, Wan Norhamidah Wan Ibrahim, Che Azurahanim Che Abdullah, Nurul Farhana Ramlan, Khozirah Shaari, Shamarina Shohaimi, Ahmed Mediani, Nurrul Shaqinah Nasruddin, Cheol-Hee Kim, Siti Munirah Mohd Faudzi

**Affiliations:** 1 Department of Biology, Faculty of Science, Universiti Putra Malaysia, Serdang 43400, Malaysia; 2 Natural Medicines and Products Research Laboratory, Institute of Bioscience, Universiti Putra Malaysia, Serdang 43400, Malaysia; 3 Department of Physics, Faculty of Science, Universiti Putra Malaysia, Serdang 43400, Malaysia; 4 The Institute of Advanced Technology (ITMA), Universiti Putra Malaysia, Serdang 43400, Malaysia; 5 Institute of Systems Biology, Universiti Kebangsaan Malaysia (UKM), Bangi 43600, Malaysia; 6 Centre for Craniofacial Diagnostics, Faculty of Dentistry, Universiti Kebangsaan Malaysia (UKM), Kuala Lumpur 50300, Malaysia; 7 Department of Biology, Chungnam National University, Daejeon 34134, Korea; 8 Department of Chemistry, Faculty of Science, Universiti Putra Malaysia, Serdang 43400, Malaysia

**Keywords:** arsenic toxicity, zebrafish, long-term learning impairment, behaviors, metabolomics

## Abstract

Arsenic trioxide (As_2_O_3_) is a ubiquitous heavy metal in the environment. Exposure to this toxin at low concentrations is unremarkable in developing organisms. Nevertheless, understanding the underlying mechanism of its long-term adverse effects remains a challenge. In this study, embryos were initially exposed to As_2_O_3_ from gastrulation to hatching under semi-static conditions. Results showed dose-dependent increased mortality, with exposure to 30–40 µM As_2_O_3_ significantly reducing tail-coiling and heart rate at early larval stages. Surviving larvae after 30 µM As_2_O_3_ exposure showed deficits in motor behavior without impairment of anxiety-like responses at 6 dpf and a slight impairment in color preference behavior at 11 dpf, which was later evident in adulthood. As_2_O_3_ also altered locomotor function, with a loss of directional and color preference in adult zebrafish, which correlated with changes in transcriptional regulation of *adsl*, *shank3a*, and *tsc1b* genes. During these processes, As_2_O_3_ mainly induced metabolic changes in lipids, particularly arachidonic acid, docosahexaenoic acid, prostaglandin, and sphinganine-1-phosphate in the post-hatching period of zebrafish. Overall, this study provides new insight into the potential mechanism of arsenic toxicity leading to long-term learning impairment in zebrafish and may benefit future risk assessments of other environmental toxins of concern.

## 1. Introduction

Among the various organs affected by bioaccumulation of heavy metals, brain damage is of particular concern due to its high susceptibility to environmental chemicals [1]. Exposure to heavy metals during neurodevelopment is believed to cause more types of neurodevelopmental disorders (NDDs) than in adulthood. However, the complexity of etiological pathways makes elucidation of this mechanism challenging [2]. The effects of environmentally relevant concentrations of heavy metals on the nervous system are usually slow-onset, irreversible, and often overlooked due to non-teratogenic effects. Children with NDDs have difficulties with sensory and motor function, communication, learning, and memory. NDDs include intellectual disabilities (ID), autism spectrum disorders (ASDs), attention-deficit/hyperactivity disorder (ADHD), and motor development disorders [3]. Impaired cognitive function interferes with normal human functioning and is often associated with neuropsychiatric disorders to varying degrees [4,5]. The global increase in NDDs [6,7] highlights the need for multidisciplinary efforts to understand the effects of low-concentration heavy metal exposure to fill data gaps in developmental neurotoxicity (DNT).

Arsenic (As) is a toxic, ubiquitous metalloid found in food, water, the environment, and various tissues of the human body [8] and has become a global health concern. The double-edged potential of As became a topic of discussion when the US Food and Drug Administration (FDA) approved arsenic trioxide (As_2_O_3_) for the treatment of acute promyelocytic leukemia (APL) in 1996 [9]. This biologically active form of As further pollutes the environment via patient excretions, as well as improper waste manufacturing and disposal [10]. Impacts during early life development are determined by the dynamics of As transit through the placental barrier, which regulates important aspects of embryonic development [11]. Exposure during pregnancy has been associated with neurological impairments in the prenatal, perinatal, and childhood periods [12]. Although it is not yet clear whether ingestion of drinking water contaminated with As at low concentrations affects children’s brains, epidemiological studies have indicated that cognitive deficits associated with As exposure [13,14] may have irreversible cumulative adverse effects years later [15]. A recent global As risk assessment predicted that 85–90% of people particularly who live in south Asia, are potentially exposed to high levels of As in groundwater from their domestic water supply [16], even at low concentrations [17]; thus, it is critical to understand the mechanism of action of As toxicity. The changing landscape, ponds, and extensive use of pesticides on palm oil plantations in the Langat Basin, Malaysia, are considered to be the major sources of increased arsenic concentration in the Langat River (0.98–21.94 µg/L), which exceed the Malaysian Ministry of Health (MOH) and World Health Organization (WHO) permissible limit for arsenic of 10 µg/L [18].

To reduce costs and shorten the duration of basic toxicity DNT studies, zebrafish (*Danio rerio*) have attracted considerable interest due to their practical benefits, including high fecundity, small size, short embryonic period, high permeability to small molecules, and transparency, which facilitate their anatomical characterization [19]. The zebrafish is universally used by biologists to study brain development [20], as it has key pathways relevant to human disease [21] with conserved gene expression for neurodevelopment [22] and brain homology [23]. Zebrafish also exhibit behavioral responses similar to those of rodents when exposed to toxicants [24], along with a behavioral repertoire that can be automatically quantified as a valuable indicator of altered brain function.

In this study, zebrafish were used as a model organism to understand the developmental effects of embryonic exposure to As_2_O_3_. To assess the long-term toxic effects of As_2_O_3_ at the functional level, motor activity, anxiety, and learning behavior were measured at different life stages. With the aim of deciphering the neurochemical changes upon exposure to As_2_O_3_ at environmentally relevant concentrations, zebrafish behavioral analysis, untargeted liquid chromatography–mass spectrometry (LCMS)-based metabolomics, and gene expression analyses were performed.

## 2. Materials and Methods

### 2.1. Zebrafish Husbandry

All experiments were performed in accordance with the Institutional Animal Care and Use Committee of Universiti Putra Malaysia (UPM) (UPM/IACUC/AUP-R049/2019), with an approval date of 23 July 2019. Adult wild-type zebrafish were maintained in freshwater at the Natural Medicines and Product Research Laboratory (NaturMeds), Institute of Bioscience, UPM. Fish were maintained at 25–27 °C with a light cycle of 14 h light: 10 h dark [25] to induce the reproductive cycle of the fish. Fertilized eggs were collected 30 min after lights were turned on. The collected eggs were incubated at 28 °C in an egg buffer solution [26]. Only embryos with intact chorionic membranes that had reached the gastrulation stage (50% epiboly) were chosen [27]. All dead or unfertilized eggs were removed. After behavioral assessment at age 6 dpf, surviving larvae were fed formulated diets and brine shrimp (*Artemia salinaa*) twice until the adult stage [28]. A total of 90 of the 6 dpf larvae from each group were evenly distributed to a different 3 L freshwater aquarium equipped with dripped water. At 14 dpf, larvae were provided with a small stream of circulating water [29] and reared in this system until the adult stage (3 months).

### 2.2. Chemical Exposure

The selected As concentrations correspond to the As concentrations reported in the domestic water supply [16]. A stock solution of 100 mM As_2_O_3_ (≥99.99%, Sigma-Aldrich, St. Louis, MO, USA) was diluted in 1 M NaOH (0.01%), which then further diluted to final concentrations of 20, 30, 40, and 50 μM in egg buffer solution. Zebrafish embryos were exposed to a varying range of As_2_O_3_ from 5 hpf to 72 hpf under semi-static conditions. For behavioral, biochemical, and metabolomics analysis, we chose a 30 μM As_2_O_3_ concentration, which does result in any morphological abnormalities at 5 hpf under semi-static conditions until hatching (72 hpf). All experiments were performed in triplicate and repeated at least three times (*n* = 90 embryos per exposure group). For larval anxiety-like response assay, an anxiogenic (100 mg/L Caffeine, Sigma-Aldrich C53) or anxiolytic drug (5 mg/L Buspirone, Sigma-Aldrich B7418) was used in 6 dpf larvae [30,31]. Both caffeine and buspirone were dissolved in egg buffer solutions at the selected concentration. Larvae were treated with caffeine or buspirone 2 h prior to behavioral recording and maintained in the same solutions during the behavioral recording [32]. To investigate learning behavior at 11 dpf, zebrafish larvae were exposed to cognition-impairing MK-801 as a positive control (M107, Sigma-Aldrich, St Louis, MO, USA). MK-801 was dissolved in sterilized water to prepare a 10 mM stock solution. The MK-801 working solution was freshly diluted from the concentrated stock solution with egg buffer solution to a final concentration of 200 μM 4 h before the experiments [33].

### 2.3. Embryonic Toxicity Test

At 4 hpf, normal fertilized embryos with intact chorion membranes were selected prior to As_2_O_3_ exposure. A comprehensive toxicity assessment was performed for each As_2_O_3_-exposed zebrafish group, including mortality rate, morphological deformities, survival to adult stage, heartbeat (count/minute), and percentage of hatching rate. The morphological deformities after As_2_O_3_ exposure in zebrafish included impairment of fin folds and tail primordium; body axis curvature (kink in tail, lordosis, or scoliosis); and abnormal shape of yolk, heart, and eyes. Body length, swim bladder diameter, and swim bladder volume of surviving larvae were measured at 6 dpf. Swim bladders were observed with an SMZ-745T stereomicroscope (Nikon, Nikon Instruments Inc., New York, NY, USA). Swim bladder volume was measured as follows: 4/3 πab^2^, (a) major horizontal axis and (b) minor vertical axis [34]. Image analysis was performed using the freely available ImageJ software (version 1.48, Wayne Rasband, National Institutes of Health, Bethesda, MD, USA from http://rsb.info.nih.gov/ij/web page (accessed on 15 July 2021). The swim bladder elongated anterior–posterior was flattened, resulting in decreased volume. As_2_O_3_-exposed embryos were raised to adult stage, and their survival was recorded throughout the growth phase.

### 2.4. Assessment of Locomotor, Anxiety, and Color Preference in Zebrafish

After As_2_O_3_ exposure, we examined locomotor activity and anxiety-like responses at the larval stage 6 dpf. Vertical swimming behavior was defined as the ability of larvae to reach the water surface [34]. Down preference in this assay denotes the percentage of larvae positioned at the bottom of the glass cylindrical column. To further examine the effect of swim bladder changes on vertical swimming behavior, we measured swim bladder diameter and swim bladder volume of larvae 6 dpf after the vertical swimming behavior was recorded. The exploratory activity of larvae was examined using an open field test that measured the distance traveled [35]. For anxiety-like response assay, we measured the percentage of down and edge preference, swimming speed, and percentage of rest. All larval behavioral procedures were performed in triplicate and repeated at least three times (*n* = 90 embryos per exposure group). An aversive stimulus (Figure S3) represented by a red moving ball was introduced to 6 dpf larvae for 5 min using Microsoft PowerPoint (version 2010, Robert Gaskins and Dennis Austin, Santa Rosa, CA, USA) [26]. ImageJ and Microsoft Excel were used to auto-generate the percentage of down and edge preference, swimming speed, and percentage of rest [32]. Assessment of avoidance response (down preference) in the anxiety-like response assay represented the percentage of larvae positioned at the bottom part of the well, as the aversive stimulation from left to right. Larval color preference was assessed in zebrafish larvae at 11 dpf by percentage of exploration maze and color preference (yellow, green, blue, and red represented by total distance traveled) [36,37,38], whereas exploratory test and color preference (green or red) were assessed in adult zebrafish at 3 months of age [39]. A summary of the behavioral assessments is provided in Supplementary Materials (Figures S1–S3A).

### 2.5. Fourier Transform Infrared Spectroscopy (FTIR)

The 6 dpf zebrafish larvae from both control and As_2_O_3_-exposed groups were fixed with 4% paraformaldehyde (PFA) and washed three times with phosphate-buffered saline (PBS) for 5 min each. All 180 larvae (90 = control, 90 = As_2_O_3_-exposed) were dried in a lyophilizer (Benchtop Freeze Dryer Labconco, Kansas City, MO, USA) at 50 °C for 12 h to remove the water content in the samples before grinding in an agate mortar and pestle to obtain zebrafish larvae powder. Larvae powder was completely mixed with dried potassium bromide (100 mg) and subjected to a pressure of 5 t in an evacuated disc for 5 min to produce a clear, transparent KBr disc with a diameter of 13 mm and a thickness of 1 mm for use in FT-IR analyses [40]. FT-IR analyses of the freeze-dried samples were performed using a Thermo Nicolet Nexus Smart Orbit spectrometer (Ramsey, NJ, USA). The spectra were recorded in the middle infrared (IR) region (500–4000 cm^−1^, in triplicate for each sample).

### 2.6. LC-MS Analysis and Metabolomics

At the end of the behavioral assessment, pools of 90 zebrafish larvae at 6 dpf were extracted; the freeze-dried tissues were homogenized in 300 µL of extraction solvent (80:20 *v*/*v*, cold methanol/water) in a 2 mL Eppendorf tube. All samples were analyzed as a single batch in random order to minimize analytical error and subjective interference and to minimize column retention shift. UHPLC analysis was performed using a Bruker impact II quadrupole time-of-flight (QTOF)–mass spectrometry system (Bruker Daltonics, Bremen, Germany) equipped with an electrospray ionization source (ESI). Chromatographic separations were performed in an Inertsil phenyl-3 column (150 × 4.6 mm with a particle size of 5 µm) (GL Sciences Inc., Rolling Hills Estates, CA, USA) for positive- and negative-ion analyses [41]. The injection volume was 10 µL, with filtration using a 0.22 µm hydrophobic PTFE membrane at a flow rate of 0.4 mL/min. The mobile phases consisted of water with 0.1% formic acid (solution A) and methanol with 0.1% formic acid (solution B). The elution gradient at 50 °C was as follows to ensure improved repeatability between runs: (1) 5% solution B for 1 min, (2) 5–50% solution B for 11 min, (3) 100% solution B for 23 min, (4) new 100% solution B for 35 min, and (5) 5% solution B for 37–50 min. The acquisition time for time-of-flight (TOF) mass spectrometry (MS) was 0.25 s, with a scan range of 70–1250 Daltons (Da). The collision energy was set to 35 V, with a collision energy spread of 15 V. A summary of sample preparation and LCMS data analysis [42,43,44,45] is provided in Figures S3B and S4 in the Supplementary Materials.

### 2.7. Quantitative Expression Analysis (qPCR)

To investigate the effects of As_2_O_3_ exposure on the expression of ASD-associated genes (adenylosuccinate lyase (*adsl*), SH3 and multiple ankyrin repeat domains 3A (*shank3a*), and tuberous sclerosis complex 1 (*tsc1b*) [46,47]), qPCR was performed in triplicate on 6 dpf zebrafish larvae (*n* = 30 larvae per sample). After chemical exposure and behavioral recording at 6 dpf, the larvae were transferred into a 1.5 mL centrifuge tube and flash-frozen for euthanization purposes. Total RNA was extracted using an RNeasy UCP micro kit (QIAGEN, Hilden, Germany, 2019), with concentration and quality checked with an ND-1000 spectrophotometer (NanoDrop Technologies, Wilmington, ED, USA). During the RNA extraction, genomic DNA was selectively removed with the clearing agent that was included in the purification kit. cDNAs were synthesized by reverse transcription using a ReverTra AceTM qPCR RT master mix with gDNA Remover (Toyobo, Japan). The cDNA concentrations were also measured using an ND-1000 spectrophotometer. The samples were then diluted with purified water, followed by the addition of 2 µL of 4× DN master mix incubated at 35 °C for 5 min. A control experiment without RNA was used to validate whether amplicons originate from cDNA and/or genomic DNA. We used *β-actin* as a reference housekeeping gene. The sequence of primers for the target genes and reference gene (*β-actin*) for zebrafish are shown in Table S1 [48]/Target genes were amplified using a CFX96 real-time PCR detection machine (Bio-Rad Laboratories, Hercules, CA, USA). The PCR reaction mixture (total 20 μ/L) contained 10 μL of SensiFAST™ SYBR No-ROX kit master mix (Meridian Bioscience, Cincinnati, OH, USA), 0.8 μL of each forward and reverse primer (10 μM), 8.4 μL of purified PCR-grade water, and 0.8 μL of cDNA sample. The thermal cycle profile was as follows: preincubation at 95 °C for 2 min; 40 cycles of amplification at 95 °C for 5 s and 60 °C for 20 s; and annealing at 65 °C for 10 s and 72 °C for 10 s. Variations in target gene expression were normalized by using β-actin expression as a reference. Delta delta Ct values (ΔΔCt) were used to calculate the relative level of gene transcription. The Ct value was determined to calculate ΔCt by subtracting the Ct value of the treated and control samples. The ΔΔCt value was obtained by subtracting the ΔCt value of the target gene of the treated sample from the ΔCt value of the housekeeping gene. The expression value of each gene was represented by the fold changed, which was calculated as follows: (x = 2^−ΔΔCt^).

### 2.8. Statistical Analysis

All experiments were repeated three times and performed in triplicate. Data were analyzed with SPSS statistical analysis software (version 22.0, IBM Corp., Armonk, NY, USA) using the probit analysis statistical method. The LC_50_ values (with 95% confidence limits) were calculated. Differences among the results were considered to be statistically significant when the *p* value was <0.05. MS Excel 2007 was used to determine the regression equation (Y = mortality; X = concentrations), and the LC_50_ was derived from the obtained best-fit line. One-way ANOVA followed by post hoc Tukey test and two-way ANOVA followed by Duncan’s test and t test were applied to determine significant differences in teratogenicity, behavior, learning impairment assessment, and gene expression between exposed and control groups. Data are presented as mean values ± standard error of the mean (SEM), with significant differences relative to the control (*p*-values ≤ 0.05). GraphPad Prism statistical software (GraphPad Software, San Diego, CA, USA) was used for all graphs.

## 3. Results

### 3.1. Developmental Toxicity Effects of Embryonic Exposure to As_2_O_3_

**Figure 1:**
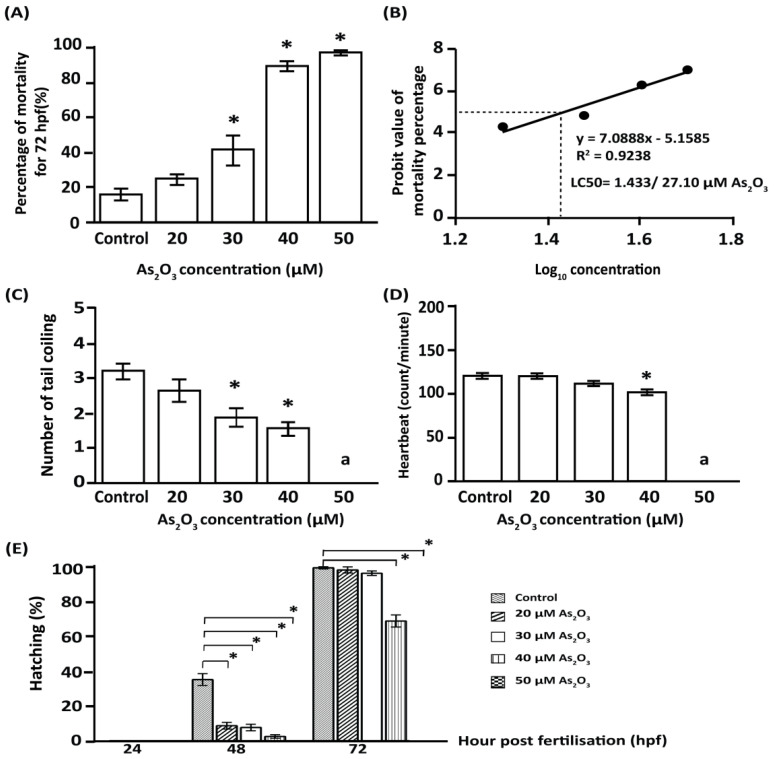
The toxicity effects of As_2_O_3_ on zebrafish (Danio rerio) embryos. (**A**) Exposure to As_2_O_3_ increased embryo mortality in a dose-dependent manner. (**B**) LC_50_ for As_2_O_3_. (**C**) Exposure to 30 µM and 40 µM As_2_O_3_ significantly decreased the incidence of tail coiling in 24 hpf embryos. (**D**) Exposure to 40 µM As_2_O_3_ significantly decreased the heartbeat of embryos examined at 48 hpf. (**E**) Exposure to increased As_2_O_3_ concentrations significantly delayed hatching between 48 hpf and 72 hpf. Data are presented as mean ± SEM of triplicate wells (*n* = 90 embryos per exposure group), with significant differences relative to the control group. ∗ (*p* ≤ 0.05); ^a^ no tail coiling, and heartbeats were recorded for 50 µM As_2_O_3_ exposed embryos, as all embryos were dead at 24 hpf.

The total percentage of mortality was represented by dead embryos that exhibited coagulation, lack of somite formation, non-detachment of the tail, and no heartbeat. Figure 1A shows the percentage of mortality in zebrafish larvae until hatching (24–72 hpf). The mortality in as-exposed embryos was increased in a dose-dependent manner. Exposure to concentrations equal to or greater than 30 µM resulted in a significant increase in mortality; in contrast, exposure to 20 µM As_2_O_3_ showed no significant difference compared to the control group. The lethal concentration (LC_50_) of As_2_O_3_ killing 50% of zebrafish embryos at 96 hpf was 27.10 µM, as shown in Figure 1B. Furthermore, no severe morphological malformations (scoliosis, yolk sac edema, or tail kinks) were observed in the exposed larval groups throughout the exposure period. Exposure to 30 and 40 µM As_2_O_3_ resulted in a decrease in the incidence of tail coiling compared to the control group (Figure 1C), whereas no tail coiling occurred in embryos exposed to 50 µM As_2_O_3_, as all embryos were dead after 24 hpf.

At 48 hpf, heartbeat was significantly decreased in larvae exposed to 40 µM As_2_O_3_, whereas no significant alterations in heartbeat were observed at lower concentrations when compared with the control group (Figure 1D). However, no heartbeat was recorded in larvae exposed to 50 µM As_2_O_3_, as all larvae were dead at 24 hpf. As shown in Figure 1E, the percentage of hatched fish decreased over time in As_2_O_3_-exposed embryos. Furthermore, 48 h of exposure to 20 µM (8.9 ± 2.2%) to 40 µM (2.8 ± 2.2%) As_2_O_3_ inhibited embryo hatching by up to 75% compared to the control group (35.1 ± 2.2%). A similar significant trend was also observed after 72 h with 40 µM (68.3 ± 2.2%) and 50 µM (0.1667 ± 2.188%) As_2_O_3_ exposure compared to the control group (98.5 ± 2.2%). In contrast, at 72 h, no significant differences in hatching were observed in 20 µM (97.3 ± 2.2%) and 30 µM As_2_O_3_ (95.4 ± 2.2%)-exposed larvae, suggesting that delaying the hatching of zebrafish embryos in the presence of As_2_O_3_ may result in abnormal organ function in subsequent developmental stages, reducing their ability to survive to adulthood. To better understand the toxic effects of As, 30 µM As_2_O_3_-exposed larvae were selected due to the significant effects of this concentration on total toxicity levels, survival to adulthood, and similarity to the arsenic concentration found in Langat Basin, Malaysia (0.98–21.94 µg/L) [18].

### 3.2. Effects of Embryonic As_2_O_3_ Exposure on Anxiety-Related Responses in 6 dpf Larvae

To further understand the toxic effects of As_2_O_3_ exposure on zebrafish larval development, we examined their anxiety-related behavior. Assessment of anxiety-related responses in this study included percent of edge preference (thigmotaxis), down preference (avoidance response), rest, and speed upon aversive stimulation. Aversive stimulus was represented by a red moving ball from left to right displayed in Microsoft PowerPoint, whereas no stimulus was represented by a blank background. As_2_O_3_ exposure resulted in no changes in anxiety-related responses in 6 dpf larvae under either condition (without/with aversive stimulus) (Table 1 and Figure S3A in Supplementary Materials, respectively) when compared to controlled larvae.

Exposure to anxiogenic caffeine increased edge preference under both conditions, reduced downward preference, reduced swimming speed, and increased rest. These results suggest that caffeine exaggerated edge preference and reduced larval avoidance behavior compared to control larvae. In contrast, exposure to anxiolytic buspirone decreased edge preference and increased down preference and swimming speed, and no resting larvae were detected, indicating that all larvae moved under both conditions compared to the control group. These results suggest that buspirone had minimal effects on larval edge preference and significantly enhanced larval avoidance behavior. Overall, the data show that As_2_O_3_, buspirone, and caffeine each have different effects on larvae.

### 3.3. As_2_O_3_ Affects Survivability and Induces Behavioral Defects during Juvenile to Adult Stages

**Figure 2:**
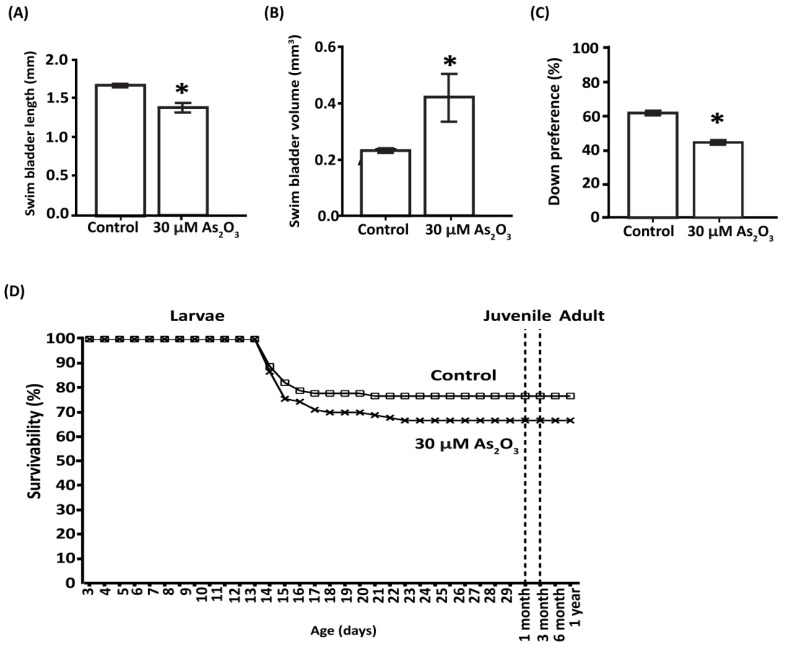
Effects of embryonic As_2_O_3_ exposure in 6 dpf larvae and adult zebrafish. At 6 dpf, As_2_O_3_ exposure increased swim bladder inflation/volume (**B**), although its anterior–posterior length was shortened (**A**). (**C**) With change in swim bladder volume, As_2_O_3_ exposure also affected swimming behavior, with reduced down preference. (**D**) In adults, embryonic As_2_O_3_ exposure resulted in a reduced percentage of survivability in As_2_O_3_ exposed larvae (x), which was mainly detected at 13–23 dpf, compared to the control group (□). Data are presented as mean ± SEM, (∗ *p* ≤ 0.05) *n* = 30–90 per group), with significant differences relative to the control group ∗ (*p* ≤ 0.05).

After the exposure period, zebrafish larvae were rinsed and reared to maturity under normal laboratory conditions. The long-term deleterious effects of As_2_O_3_ were evidenced by reduced survivability at 72 hpf, increase in swim bladder volume with impaired vertical swimming behavior at 6 dpf, and a persistent decrease in exploratory behavior until adulthood. However, increased swim bladder volume did not affect survivability itself at 72 hpf after As_2_O_3_ exposure. For both control and As_2_O_3_-treated larvae, survival was 100% by day 12 and decreased to 86.7% by day 13 (Figure 2D). The survivability was maintained after day 14 in control and after 23 dpf in As_2_O_3_-exposed fish during juvenile and adult growth. Throughout the rearing process, not a single death was recorded after 72 hpf in As_2_O_3_-exposed fish, comparable to the control group. Exposure to As_2_O_3_ (1.4 ± 0.05 mm^3^) resulted in a smaller diameter of the posterior lobe of the swim bladder versus the control group (1.7 ± 0.05 mm^3^, Figure 2A). This was supported by the fact that swim bladder volume was significantly enlarged in As_2_O_3_-exposed larvae (0.4 ± 0.07 mm^3^) compared to that of the control group (0.3 ± 0.07 mm3, Figure 2B). Increased swim bladder volume affected the neutral buoyancy of larvae, ultimately affecting their survival in later stages, as well as subsequent behavior. Larvae exposed to As_2_O_3_ (44.6 ± 1.1%) showed a significantly decreased down preference when compared with the control group (61.8 ± 1.1%) (Figure 2C). These excessive floating attempts were consistent with the higher swim bladder volume observed in As_2_O_3_-exposed larvae, indicating an impaired control of neutral buoyancy affecting swim behavior [34].

**Figure 3:**
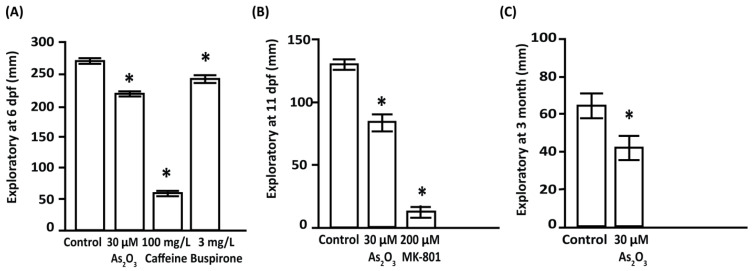
Effects of long-term impairment of 30 µM As_2_O_3_ exposure on exploratory activity. Exploratory activity was persistently decreased in larvae from 6 dpf (**A**) and 11 dpf (**B**) to adult stage (**C**). Data are presented as mean ± SEM, (∗ *p* ≤ 0.005), *n* = 30 larvae per group, *n* = 22 adults per group), with significant differences relative to the control group, ∗ (*p* ≤ 0.05).

As_2_O_3_ caused a persistent reduction in larval exploratory behavior until adulthood. The exploratory behavior of zebrafish at 6 dpf, represented by the average distance traveled, was significantly decreased in As_2_O_3_-exposed larvae (219.6 ± 5.7 mm) compared to the control group (271.7 ± 5.7 mm, Figure 3A). These findings are consistent with the positive control, caffeine-treated larvae (58.1 ± 5.7 mm), which showed a significant reduction in average distance traveled. However, buspirone treatment (negative control) resulted in a longer average distance traveled (242.2 ± 5.7 mm), which was significantly greater than that of the other exposed groups. The exploratory activity of zebrafish was further evaluated at 11 dpf and 3 months of age. The results showed that exploratory activity of As_2_O_3_-exposed larvae (83.6 ± 12.8 mm) was significantly reduced compared to control larvae (131.4 ± 12.8 mm Figure 3B). Coincidentally, zebrafish larvae that were treated with cognitive impairer MK-801, which mechanistically blocks NMDA/glutamatergic signaling, showed a severe reduction in exploratory activity (11.4 ± 12.8 mm) compared to control larvae (Figure 3B). In addition, no morphological deformities were observed in MK-801-exposed larvae throughout the exposure period. Locomotor behavioral assessment, represented as exploratory activity, performed prior to any aversive or color stimulation showed that these detrimental effects persisted into the adult stage, as As_2_O_3_ (417.7 ± 91.8 mm) significantly decreased exploratory activity compared to control larvae (640.5 ± 91.8 mm) (Figure 3C).

### 3.4. As_2_O_3_ Exposure Affects Color Preference and Learning Impairment

**Figure 4:**
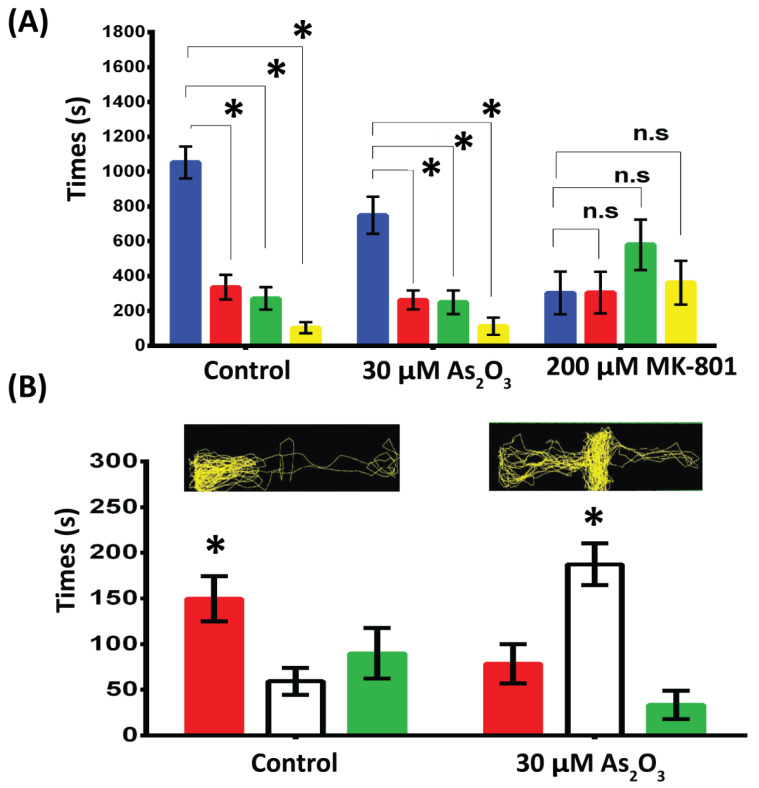
Effects of embryonic As_2_O_3_ exposure on the color preference of larval (11 dpf, **A**) and adult zebrafish (**B**). (**A**) In the 11 dpf larval test, a cross maze with four different-colored cambers was used. Reduction in color preference for blue in As_2_O_3_-exposed larvae compared to the control group. No significant differences were observed in color preference for red and green in control or As_2_O_3_-exposed larvae. However, MK-801-exposed larvae showed no clear color preference (**B**) Five-minute video tracking of color preference in adult fish after associative learning. Adult fish were acclimated to the three-chamber maze for 6 days with red food association before the test on the 7th day. As_2_O_3_-exposed zebrafish showed no significant preference for either green or red color Data are presented as mean ± SEM, (∗ *p* ≤ 0.005). *n* = 30 larvae per group, *n* = 22 adults per group. ∗ Significance at *p* ≤ 0.05 between left/center/right and between two color arms for each test, n.s: not significant.

An innate color preference test was performed in a plus maze with four different-colored sleeves to assess the effects of embryonic As_2_O_3_ exposure in 11 dpf zebrafish larvae. This color test showed that control zebrafish larvae exhibited distinct color discrimination and color preference. We observed a reduction in color preference for blue in As_2_O_3_-exposed larvae (749.3 ± 132.4 s) compared to the control group (1052 ± 133.5 s) (Figure 4A). No significant differences were observed in color preference for red and green in control (336.1 ± 132.4 s vs. 271.6 ± 132.4 s) or As_2_O_3_-exposed larvae (262.7± 132.4 s vs. 250.2 ± 132.4 s). However, MK-801-exposed larvae showed a change in color preference (blue: 303.3 ± 132.4 s, red: 305.3 ± 132.4 s, green: 579.8 ± 132.4 s, and yellow: 361.6 ± 132.4 s). MK-801-treated larvae swam longer in the center of the maze without showing a clear color preference.

Because the color preference test was previously used to evaluate adult zebrafish behavior for learning and memory [49,50], we examined color preference in 3-month-old adult zebrafish using a three-chamber apparatus with red and green sleeves to assess long-term effects of embryonic As_2_O_3_ exposure on color preference in adult fish. It was previously established that red is the most preferred color in zebrafish and that associations with red are easily learned from food color (for example, brine shrimp) during rearing [51]. Thus, we used red color preference for food-associative learning in adult zebrafish. To increase food color-associated learning in the color preference test, adult fish were acclimated in the maze for 6 days with red color brine shrimp feeding before the test on the 7th day. In the three-chamber color preference test, control fish showed a tendency to prefer the red-colored zone over the green zone (red, 139.1 ± 22.0 s; green, 78.5 ± 22.0 s; center, 81.9 ± 22.0 s). However, no such response was observed in the As_2_O_3_-exposed adult group, showing no significant preference for either green or red color (red, 74.6 ± 22.0 s; green, 62.2 ± 22.0 s; center: 162.6 ± 22.0 s) (Figure 4B). This lack of color preference and lost directional preference (Figure S12) in the adult stage may indicate an association with color impairment in the As_2_O_3_-exposed group.

### 3.5. Disturbance of Lipid and Fatty Acid Metabolites

To assess changes in biochemical information and understand both molecular structure and molecular composition, freeze-dried samples of 6 dpf control (whole body) and 30 µM As_2_O_3_-treated zebrafish larvae were subjected to FTIR analysis. As_2_O_3_ exposure causes biochemical alterations in proteins, lipids, carbohydrates, and nucleic acids of larvae. The regions of transmittance in the FTIR spectra are directly proportional to concentration of the molecules. The FTIR spectrum of 6 dpf zebrafish larvae showed a complex of several bands originating from functional groups belonging to lipids, proteins, nucleic acids, and carbohydrates (Table S2 and Figure S6 in Supplementary Materials). However, a more detailed picture of altered metabolites could be identified using a more sophisticated LCMS-based metabolomics tool to detect a broad spectrum of affected metabolites with high sensitivity and resolution.

**Figure 5:**
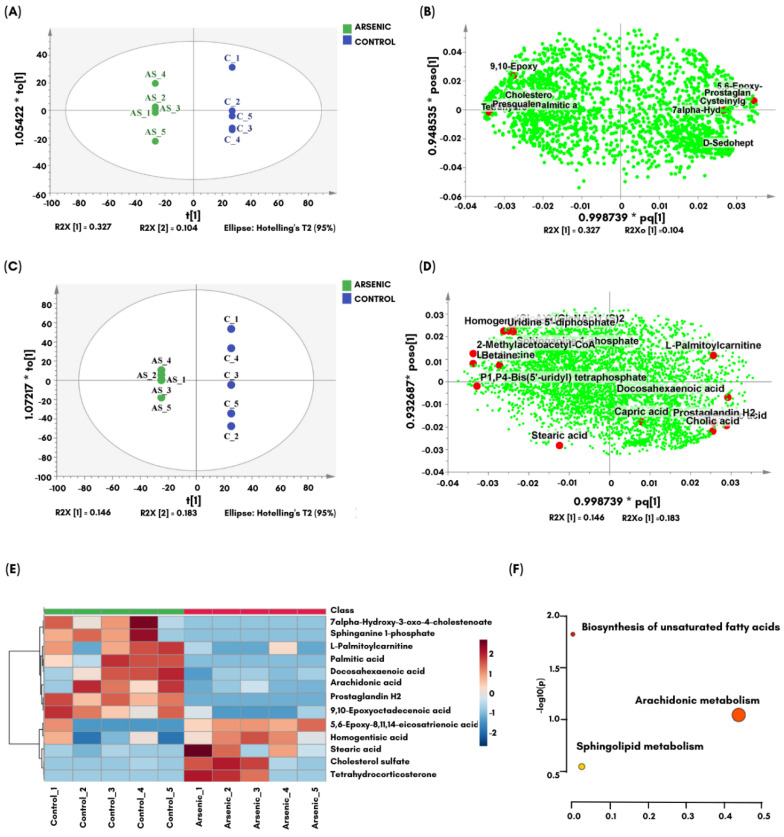
OPLS−DA score plot and loading scatter plot (**A**,**B**) of negative and positive (**C**,**D**) modes based on the zebrafish larvae normalized data exposed to 30 µM As_2_O_3_ in comparison to control larvae. (**E**) Differential expression of metabolites in As_2_O_3_-exposed larvae produced by hierarchical clustering of the most significantly upregulated (red) and downregulated (blue) metabolites obtained from in negative- and positive-ion modes compared to the control group based on the log2 fold change value. (**F**) Metabolic set enrichment analysis of lipid metabolites in 6 dpf zebrafish showed the biosynthesis of unsaturated fatty acids, arachidonic metabolism, and sphingolipid were dysregulated after embryonic exposure to As_2_O_3_. Color intensity (yellow–to–orange/red) represents increasing statistical significance, whereas circular diameter is related to pathway impact. The graph was obtained by plotting−log of *p*-values from pathway enrichment analysis on the *y*-axis and the pathway impact values derived from pathway topology analysis on the *x*-axis.

One approach to detect significant similarities and differences among affected metabolites in large metabolomics datasets is multivariate data analysis (MVDA) methods, including unsupervised principal component analysis (PCA) and supervised partial least squares-discriminant analysis (PLS-DA). In this MVDA method, the tested samples are clustered based on their variance by exposing them to different principal components (PCs). The metabolites are believed to be responsible for group separation by PCA (Figure S7) and PLS-DA loadings (Figure S8). To further identify the significant metabolites contributing to the discrimination, a second precise and straightforward comparison was performed between the As_2_O_3_-exposed zebrafish larval group and the control group, as shown in the supervised orthogonal projections to latent structure discriminant analysis (OPLS-DA) (Figure 5). OPLS-DA using the S-plot was helpful in clarifying and identifying biomarkers in both groups.

The potential biochemical biomarkers for the long-term effects of As_2_O_3_ compared to control larvae were further computed using a supervised OPLS-DA analysis (score and loading plots of negative (Figure 5A,B) and positive (Figure 5C,D) mode) to correlate the changes in metabolites in As_2_O_3_-exposed larvae with neurotoxicity and behavioral abnormalities. The generated OPLS-DA score plot shows significant differences between the As_2_O_3_-exposed zebrafish larvae and the control groups (Figure 5A,C). In negative mode, four components were produced (Figure S9A,B) with acceptable goodness of fit and predictability of the model, as evidenced by R2Y and Q2Y values of 0.997 and 0.912, respectively. In contrast, the positive mode generated five components (Figure S9C,D), with an R2Y value of 1 and a Q2Y value of 0.657. One hundred random permutations of the y variables confirmed the minimal validity of the current model of *y*-axis intercepts below zero, as indicated in SIMCA (Figure S11). The validity of metabolite changes was supported by observed vs. predicted plots, with excellent correlation between all features (y data) and metabolites (x data), as represented by regression lines with a value of 1 (Figures S10 and S11).

Of the total 150 identified features, 13 metabolites were found to be significantly altered between the As_2_O_3_-exposed and control groups. Table S3 lists the identified metabolites and their changes with significant *p* and FDR values. The generated *p* and FDR values are <0.01, which is below the threshold of 0.05, indicating that the observed changes are statistically robust. The log2 fold change in metabolite levels, presented as red pixels (for upregulation) and blue pixels (for downregulation) in Table S3 (in Supplementary Materials), are shown in the heatmap (Figure 5E). The results show that nine metabolites were significantly downregulated in As_2_O_3_-exposed zebrafish larvae, including arachidonic acid, docosahexaenoic acid (DHA), palmitic acid, 9,10-epoxyoctadecenoic acid, sphinganine-1-phosphate, L-palmitoylcarnitine, prostaglandin, cholesterol, and sulfate tetrahydrocorticosterone. In contrast, four metabolites, namely 5,6-epoxy-8,11,14-eicosatrienoic acid, 7α-hydroxy-3-oxo-4-cholestenoate, stearic acid, and homogentisic acid, were upregulated. Following FTIR and LCMS-based metabolomics analyses, which showed that As_2_O_3_ primarily affected lipids, further targeted data analysis of lipid metabolites was performed, which included pathway enrichment analysis combined with topology analysis to identify the major metabolic pathways affected by As_2_O_3_ (Figure 5F). Based on KEGG metabolic pathways, As_2_O_3_ was found to be responsible for disrupting three major metabolic pathways, namely unsaturated fatty acid biosynthesis, arachidonic acid, and sphingolipid metabolism in zebrafish larvae.

### 3.6. Alterations in ASD-Associated Genes

**Figure 6:**
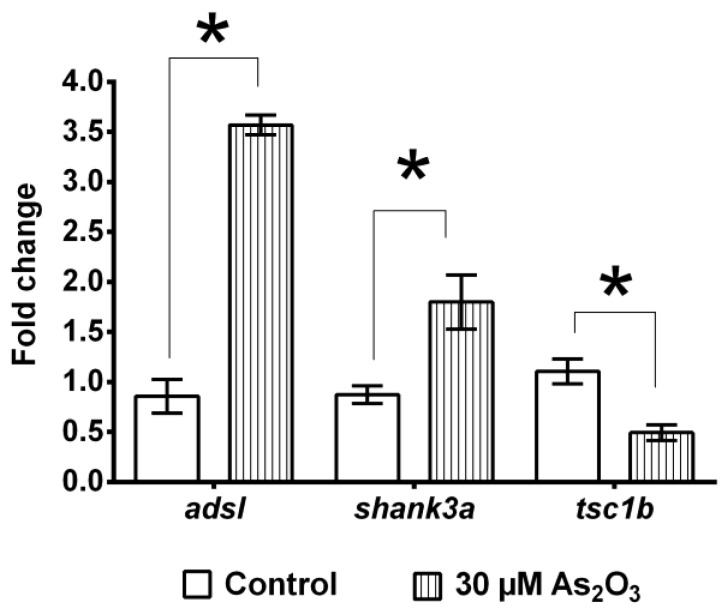
Embryonic exposure to As_2_O_3_ induced alterations in transcriptional regulation of ASD-associated genes. As_2_O_3_ exposure resulted in significant overexpression of *adsl* and *shank3a*, whereas *tsc1b* was downregulated. Data are presented as the mean ± SEM, (* *p* < 0.05) (*n* = 90 per group).

As_2_O_3_ exposure resulted in significant overexpression of *adsl* (3.57 ± 0.19) and *shank3a* (1.8 ± 0.31) genes and downregulation of tcs1b (0.49 ± 0.15) compared to control larvae (*adsl*: 0.8585 ± 0.1949, *shank3a*: 0.87± ± 0.30, *tsc1b*: 1.106 ± 0.1466) (Figure 6). Overall, the results of this study highlight that embryonic exposure to environmentally relevant As concentrations in zebrafish embryos could be associated with NDD, particularly ASD. ASD-associated genes were selected due to the widespread prevalence of ASD-like symptoms, such as cognitive deficit, and the anticipated burden of ASD in children in Malaysia. A proportion of 3.3% of infants in Malaysia were reported to exhibit developmental delays, with 4.7% of children born with disabilities [52]. Besides genetics, environmental exposure to arsenic is a potential risk factor for ASD [53], which is usually accompanied subtle effects in childhood with potentially late onset [54].

## 4. Discussion

This study demonstrated that embryonic exposure to As_2_O_3_ in zebrafish increased mortality, decreased heart rate, and reduced the incidence of tail coiling in a dose-dependent manner. Additionally, As_2_O_3_-exposed larvae showed motor behavior deficits, followed by an impairment in color preference at 11 dpf and later in adulthood. Preliminary biochemical evaluation by FTIR showed that 30 µM As_2_O_3_ induced changes in lipid, protein, carbohydrate, and nucleic acid profiles. Metabolomics analysis further revealed disruption of lipid metabolites involving arachidonic, sphingolipid, and biosynthesis of unsaturated fatty acid metabolism in As_2_O_3_-treated zebrafish larvae. By integrating metabolite dysregulation, behavioral alteration, and altered regulation of ASD-associated genes, these results support the idea that embryonic As_2_O_3_ exposure could be involved in NDD pathogenesis. Lipid alteration associated with cognitive deficit, which have been reported in both ASD and ADHD [55], highlights the possibility that a similar metabolism could be affected likewise in NDD.

Exposure to concentrations equal to or greater than 30 µM As_2_O_3_ caused a significant increase in mortality and induced weak heartbeats. This result is consistent with previous studies, which reported that zebrafish exposed to a range of As in later stages (15–96 hpf) developed edema and scoliosis, which could lead to cardiac malformations and mortality [56]. In contrast, 20–50 µM As_2_O_3_-exposed embryos (5–72 hpf) showed no morphological abnormalities. However, a significant increase in embryo mortality was observed after exposure to 40–50 µM As_2_O_3_. The increase in mortality and occurrence of abnormalities after exposure to thousand-fold As (2 mM) began prior to gastrulation (4 hpf) and post gastrulation (6 hpf), signifying the vulnerability of the exposure window [57,58]. This suggests that zebrafish larvae are more sensitive to toxic effects of As_2_O_3_ at younger ages, even at micromolar concentrations, resulting in reduced survival into adulthood, also affecting defective swimming activity [34]. As_2_O_3_ exposure also affected swim bladder inflation and vertical swimming behavior. However, further exploration is required to identify the molecular mechanism underlying swim bladder development or inflation [59,60]. Larvae stayed less on the edge after exposure to As_2_O_3_ and buspirone, suggesting that anxiety was reduced, although non-significantly. In contrast, larvae tended to stay on the edge after exposure to caffeine, suggesting that anxiety was increased. This result is consistent with previous reports that high levels of caffeine can increase anxiety [32,61] and increase edge preference in response to visual stimuli [32].

Although no noticeable malformations were observed, micromolar As_2_O_3_ revealed its toxic effect by reducing the incidence of tail coiling and causing a persistent reduction in larval locomotor activity until the adult stage. This cumulative effect suggests that embryos treated with As_2_O_3_ from the gastrulation stage could be impaired in essential neurodevelopmental processes [60,61]. However, further investigation is required to identify the exact molecular mechanism. The obtained results are comparable to those of a previous embryonic exposure study investigating thousand-fold NaAsO_2_ exposure (1 mM) (0–120 hpf), which reduced locomotor activity in zebrafish [62]. A similar trend was observed in rats, with locomotor decreased activity by 0.10 mg/L AsNaO_2_, which was associated with an increase in oxidative stress and inhibition of AChE in the striatum [63]. Another study [64] reported decreased myoblast proliferation and a reduced number of muscle fibers, resulting in a long-lasting impairment of locomotor activity in fish that persisted into adulthood [65] after exposure to 1 µM arsenite for 72 h, which is consistent with the results of the present study. However, As_2_O_3_ exposure had no effect on anxiety responses in the present study.

Innate color preferences are vital abilities for fish to learn and make decisions [51] as one such example of associative learning, which is critical for foraging and navigation [66]. Although larvae explored all available color choices, we observed a significant reduction in color preference for blue in 11 dpf zebrafish after embryonic As_2_O_3_ exposure. We also observed impairment in color preference (red > green) in adults after 3 months of embryonic As_2_O_3_ exposure. The presence of four cone photoreceptors in the zebrafish retina enables the detection of light with short wavelengths (ultraviolet, blue), which is required for non-opponent processing; and medium (green) and long (red) wavelengths, which are required for opponent processing [67]. Strong blue preference was exhibited in As_2_O_3_-exposed and control larvae due to their preference for shorter light wavelengths [68]. The preference for red is associated with food foraging [69], whereas yellow has been proposed as a warning signal that allows fish to assess potential mates or locate less common resources that contribute to avoidance behavior [70]. The yellow-zone avoidance observed in the current study was consistent with reports in previous studies [38,68].

Learning impairment became evident in adulthood. As_2_O_3_-exposed fish showed no directional or color preference, indicating an impairment in associative learning. In comparison, control zebrafish spent a longer time in the right chamber and preferred red over green color, regardless of the location of color sleeves. The rightward preference observed in control adult zebrafish was attributed to the right eye being used to view a novel environment [71]. The introduction of red pigment-enriched diets (brine shrimp, pellets) throughout the rearing period caused zebrafish to learn to associate red color with the presence of food [72]. This result demonstrates that As_2_O_3_ exposure can affect learning or eye development, which manifests later in adulthood [73,74]. Consistent with these results, arsenic exposure has been shown to decrease retina thickness and affect zebrafish eye development [75]. Thus, these findings raise the possibility that arsenic could damage retinal pigment epithelia, leading to visual disturbances and impairing the ability to detect and evaluate surrounding stimuli needed for survival.

Preliminary FTIR analysis showed that As_2_O_3_ destructively affects functional groups, such as proteins, lipids, carbohydrates, and nucleic acids, in zebrafish larvae. The decrease in intensity of C=O bands of amide at 1542.77 cm^−1^ (control) and 1538.98 cm^−1^ (As_2_O_3_-exposed) indicates a change in protein structure or protein synthesis resulting from As_2_O_3_ exposure. Protein degradation was previously reported in relation to the interaction of As with sulfhydryl groups in protein [76,77]. This toxic interaction could trigger the formation of free radicals, leading to oxidative damage, which could alter protein conformation, function, and interaction with other functional proteins, as shown in rat brains after As exposure [78]. The decrease in intensity of the asymmetric CH_3_ stretch band suggests a change in lipid content upon As_2_O_3_ exposure and might be related to increased lipolytic activity. Similar destructive effects of As_2_O_3_ were observed in the kidney tissue of freshwater fish (*Labeo rohita*), causing biochemical changes in proteins, lipids, and nucleic acids, resulting in functional deformations [79]. In addition, chronic As exposure has been reported to impair lipid metabolism and consequently decrease cognitive functions [80,81].

Further supporting the FTIR findings, LCMS-based metabolomics data show that As_2_O_3_ leads to a deficiency of essential polyunsaturated fatty acids (PUFAs) in the biosynthesis of unsaturated fatty acids, including arachidonic acid, docosahexaenoic acid (DHA), stearic acid, and palmitic acid. PUFAs are known to play a central role in mediating cognitive functions. As_2_O_3_ also showed that cyclooxygenase (COX)-associated metabolites (arachidonic acid and prostaglandin) were significantly reduced in the As_2_O_3_-treated group. This result is consistent with a study in mice, in which As increased cyclooxygenase-2 (COX-2) mRNA while decreasing prostaglandins, resulting in a decrease in arachidonic acid levels [82]. An increase in COX activity triggered an increase in inflammation in the hippocampus, leading to impaired spatial memory in mice [82], suggesting that embryonic As exposure decreases arachidonic levels, which are associated with changes in prostaglandin levels, leading to behavioral impairments. High As exposure activates microglia with a reactive proinflammatory phenotype, as well as increases in inflammatory markers, such as prostaglandins, which have been associated with memory impairment [83].

Furthermore, As_2_O_3_ disrupted sphingolipid metabolism by increasing sphingosine-1-phosphate metabolite in exposed larvae, which can lead to vascular defects and pericardial edema in zebrafish [84,85,86]. This is consistent with our results showing a dose-dependent reduction in heart rate, highlighting that upregulation of sphingosine-1-phosphate in exposed larvae plays a crucial role in their survival at later stages of growth. In addition, DHA deficiency can disrupt neuronal development, stimulate apoptosis, and increase tissue inflammation [87]. DHA deficiency has also been linked to impaired cognitive abilities and abnormal emotions [88], which may affect brain function in adulthood [89], as observed in association with persistent learning deficits in As_2_O_3_-exposed adults.

Overexpression of *adsl* and *shank3a* genes and downregulation of *tcs1b* genes suggest that behavioral impairments are associated with ASD. The upregulation of the *adsl* gene in As_2_O_3_-exposed larvae may indicate a lack of purine nucleotide production, a decrease in the purine nucleotide cycle, and an accumulation of defective enzyme substrates [90]. The accumulation of uridine and its derivatives detected in As_2_O_3_-exposed larvae also confirms that purine nucleotides and de novo synthesis of pyrimidine were impaired, accelerating the biosynthesis of pyrimidine nucleotides [91]. The alteration in the expression of synaptic scaffolding protein *shank3* detected in the present study is consistent with *shank3* mutations associated with NDD, such as ASD, ID, and schizophrenia, in several cohort studies [92,93] and with manic-like hyperkinetic behavior in transgenic mice [94,95].

Downregulation of the *tsc1b* gene in As_2_O_3_-exposed larvae may involve overstimulation of the mammalian target of rapamycin (mTORC1), leading to metabolic overactivity and excessive cell growth, causing many of the multisystem effects of *tsc* [96,97]. Alterations in negative regulators of mTORC1 have been associated with ASD, ADHD, cognitive deficits, and affective disorders [98]. Consistent with this phenomenon, mice with defective *tsc1/2* show autistic traits, such as reduced cognitive abilities, social interaction, and repetitive behaviors [99]. Therefore, the downregulation of the *tsc1* gene detected in As_2_O_3_-exposed larvae may be responsible for cognitive deficits observed in the current study. Hence, this multi-model analysis was necessary for future studies and allows for exploration of how NDD-related phenotypes may arise by confirming the refined validity of environmental and genetic factors influencing NDD risk. Taken together, these results provide evidence of an association between abnormal ASD-like genes, metabolite changes, and As exposure.

## 5. Conclusions

Embryonic exposure of embryos to low concentrations of 30 and 40 µM As_2_O_3_ significantly decreased the number of tail-coil movements, heartbeat, and swimming activity. Although no changes in anxiety-like responses were observed in larvae at 6 dpf, the toxic effects of 30 µM As_2_O_3_ were delayed and manifested in later stages of growth. The long-term embryonic effects of 30 µM As_2_O_3_ exposure were evidenced by reduced survival and delayed hatching in early larval stages, as well as alterations in motor response and loss of directional and color preference in adult zebrafish. Preliminary FTIR analysis combined with the sophisticated LCMS-based metabolomics approach showed that As_2_O_3_ exposure affected biochemical changes in proteins, lipids, and nucleic acids of larval zebrafish, particularly arachidonic acid, docosahexaenoic acid, prostaglandin, and sphinganine-1-phosphate metabolites in the post-hatching period of zebrafish. Additionally, we showed concomitant upregulation of *adsl* and *shank3a* and downregulation of *tsc1b* genes. This study shows that the integration of toxicity, behavior, metabolomics, and gene expression is a promising approach to understanding the mechanisms underlying behavioral disorders associated with NDDs. Nevertheless, an additional multi-omics approach is needed in future studies to obtain a holistic view linking the interaction between genotype and behavioral phenotypes. Overall, this study provides new clues with respect to the possible mechanism of embryonic arsenic toxicity, leading to long-term learning disorders in zebrafish and benefiting future risk assessments of arsenic and other environmental contaminants of concern.

## Figures and Tables

**Table 1:** Changes in anxiety-related responses in As_2_O_3_-, caffeine-, or buspirone-exposed larvae.

Anxiety-Related Response	Controlled Larvae		30 µM As_2_O_3_-Exposed Larvae		100 mg/L Caffein-Exposed Larvae		5 mg/L Buspirone-Exposed Larvae	
	Aversive Stimulus		Aversive Stimulus		Aversive Stimulus		Aversive Stimulus	
	Without	With	Without	With	Without	With	Without	With
Edge preference	84.0% ± 3.4	87.0% ± 3.4	83.0% ± 3.4	84.6% ± 3.4	89.9% ± 3.3	92.1% ± 3.7	73.9% ± 5.6	79.3% ± 4.8
Down preference	48.0% ± 3.9	54.0% ± 3.9	43.1% ± 3.9	49.6% ± 3.9	47% ± 2.8	58.9% ± 2.9	57.9% ± 4.1	74.3% ± 3.9
Speed	42 ± 3.1 mm/min	39 ± 3.0 mm/min	42.8 ± 3.0 mm/min	47.3 ± 3.0 mm/min	9 ± 2.1 mm/min	11.0% ± 2.1 mm/min	47 ± 2.1 mm/min	43 ± 2.1 mm/min
Rest	20% ± 4.6	24% ± 4.6	17.4% ± 3.0	18.8% ± 3.0	72% ± 2.1	74% ± 2.1	0%	0%

## Data Availability

Data are contained within the article or Supplementary Material.

## Supplementary Materials

The following supporting information can be downloaded at: https://www.mdpi.com/article/10.3390/toxics10090493/s1, Figure S1: Summary of locomotor assessment in (A) 6 dpf and exploratory activity in (B) 11 dpf larvae and (C) 3-month-old adult zebrafish (D); Figure S2: Summary of color preference assessment in (A) 11 dpf larvae and (B) 3-month-old adult zebrafish. This assay was repeated at least three times, and a total of larvae *n* = 90 and *n* = 22 adult zebrafish were tested per treatment group; Figure S3: Summary of (A) anxiety-like response and (B) sample preparation for LCMS analysis in 6 dpf larvae; Figure S4: Summary of LCMS data analysis; Figure S5: Effects of As_2_O_3_, caffeine, and buspirone on anxiety-like responses of 6 dpf zebrafish larvae; Figure S6: General band assignment of the FTIR spectra of (A) control and (B) 30 µM As_2_O_3_-exposed zebrafish larvae in the 500–4000 cm-1 regions; Figure S7: PCA score plot (A,B,E,F) and loading scatter plot (C,D,G,H) of negative and positive modes based on the normalized data of zebrafish larvae exposed to 30 µM As_2_O_3_ compared to control for non-polar and polar features; Figure S8: PLS-DA score plot (A–D) and loading scatter plot of negative modes based on zebrafish larvae normalized data exposed to 30 µM As_2_O_3_ in comparison to control for non-polar and polar features; Figure S9: PLS-DA validating models of negative and positive mode based on zebrafish larvae normalized data exposed to 30 µM As_2_O_3_ in comparison to control for non-polar and polar features; Figure S10: OPLS-DA summary of the fit of negative (A) and positive (B) modes based on zebrafish larvae normalized data exposed to 30 µM As_2_O_3_ in comparison to control; Figure S11: OPLS-DA validating models (A–D) of negative and (E–H) positive mode based on zebrafish larvae normalized data exposed to 30 µM As_2_O_3_ in comparison to control; Figure S12: Control adult zebrafish had increased preference for the right chamber compared to As_2_O_3_-exposed zebrafish, which lost directional preference; Table S1: Designated forward and reverse sequence of selected genes associated with ASD based on available references; Table S2: General band assignment of the FTIR spectra of control and 30 µM As_2_O_3_-exposed zebrafish larvae; Table S3. List of identified and significant metabolites in As_2_O_3_-exposed larvae in comparison to the control group (*p* < 0.05).
