# Supplementary material for: Embryonic Arsenic Exposure Triggers Long-Term Behavioral Impairment with Metabolite Alterations in Zebrafish
Source: Toxics. 2022 Aug 24;10(9):493. doi: 10.3390/toxics10090493 (PMC9502072; doi:10.3390/toxics10090493)
Supplement: Supplementary file 1 [file toxics-10-00493-s001.zip › toxics-1848768-supplementary.pdf]

toxics

MDPI

Supplementary Materials: Embryonic Arsenic Exposure Triggers Long-Term Behavioral Impairment with Metabolite Alterations in Zebrafish

Noraini Abu Bakar, Wan Norhamidah Wan Ibrahim, Che Azurahanim Che Abdullah, Nurul Farhana Ramlan, Khozirah Shaari, Shamarina Shohaimi, Ahmed Mediani, Nurrul Shaqinah Nasruddin, Cheol-Hee Kim and Siti Munirah Mohd Faudzi

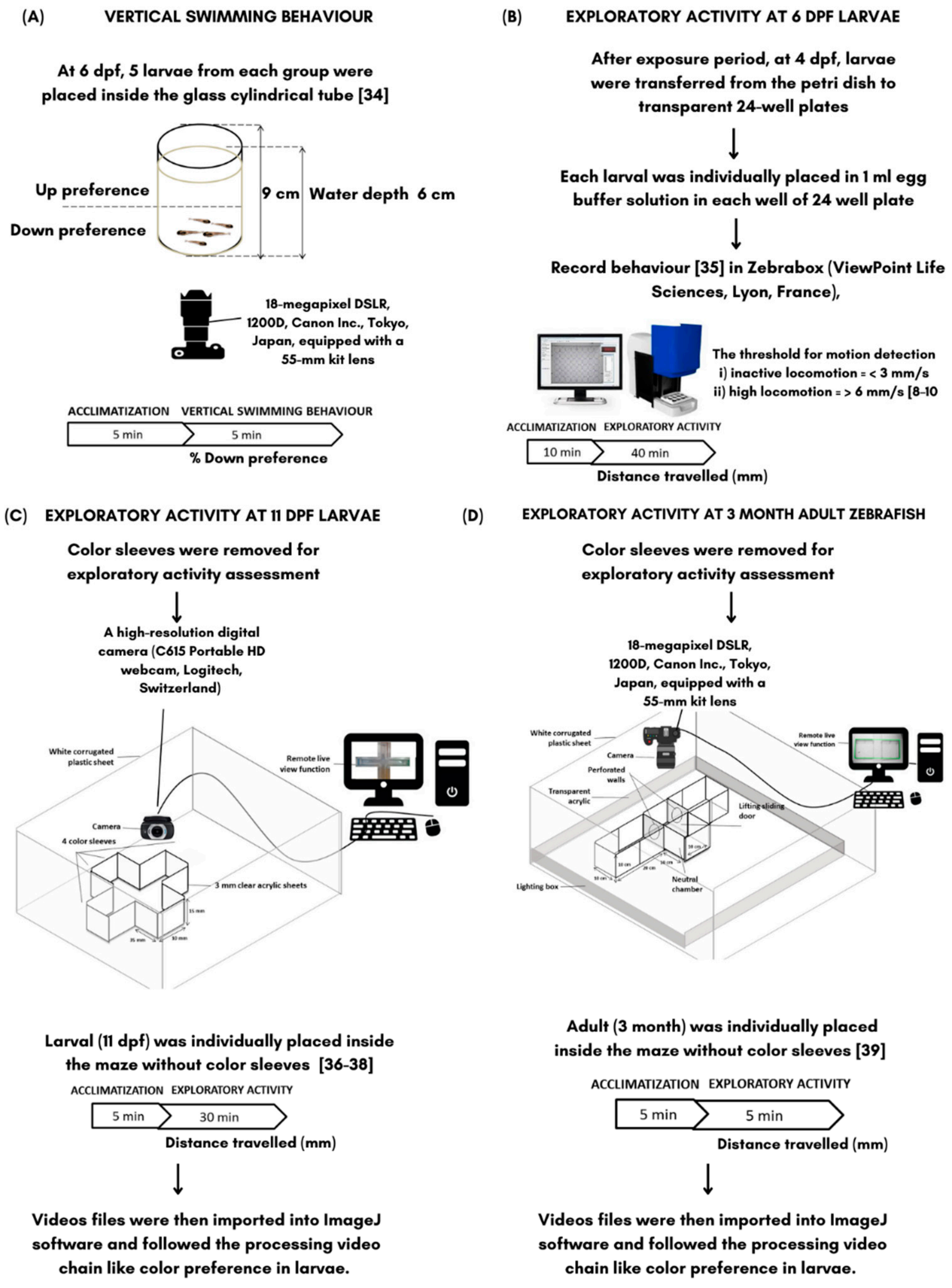

Figure S1. Summary of of locomotor assessment in (**A**) 6 dpf and exploratory activity in (**B**) 11 dpf larvae and (**C**) 3 month old adult zebrafish (**D**).

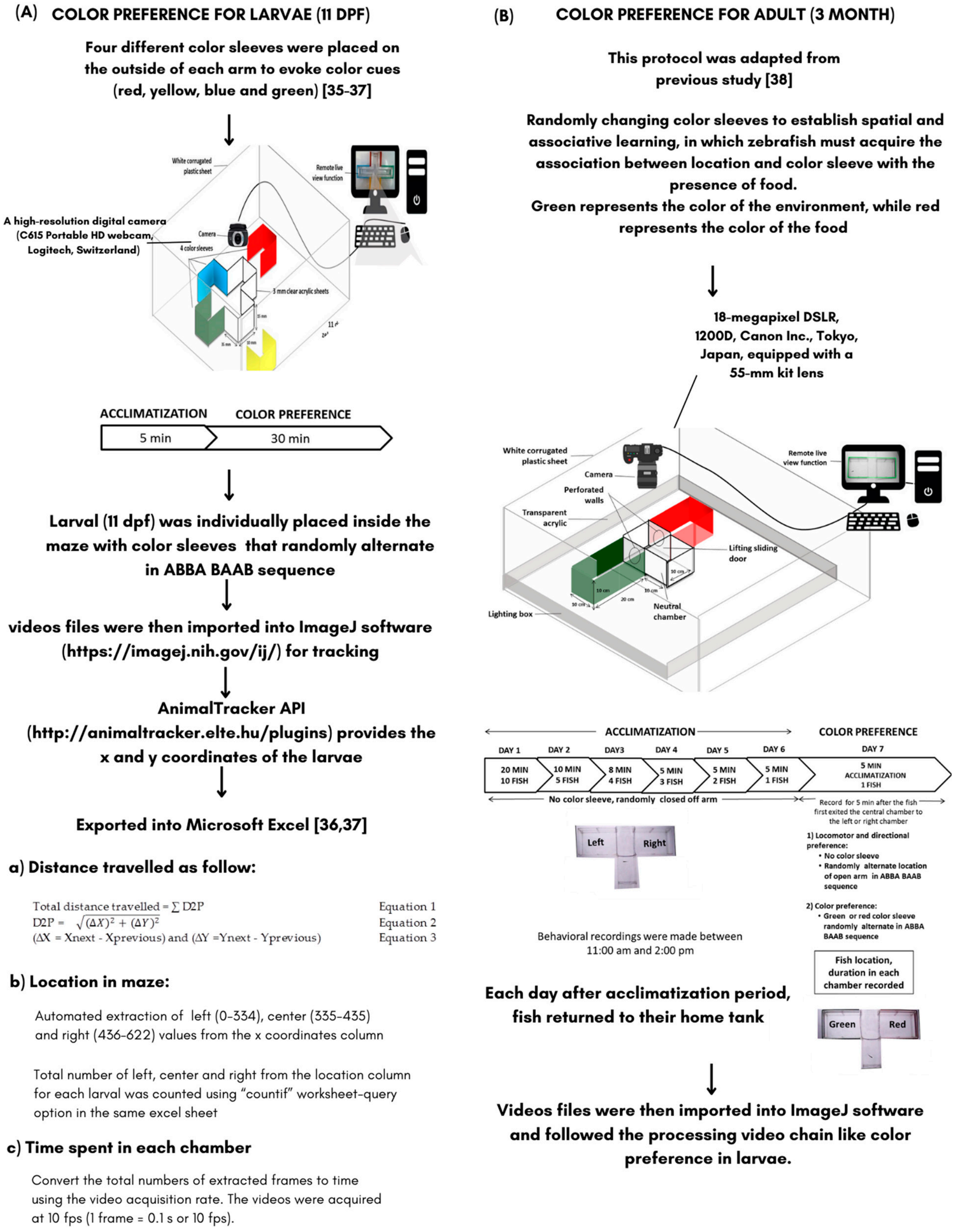

Figure S2. Summary of color preference assessment in (A) 11 dpf larvae and (B) 3 month old adult zebrafish. This assay was repeated at least three times and a total of larvae $n = 90$ and $n = 22$ adult zebrafish were tested per treatment group.

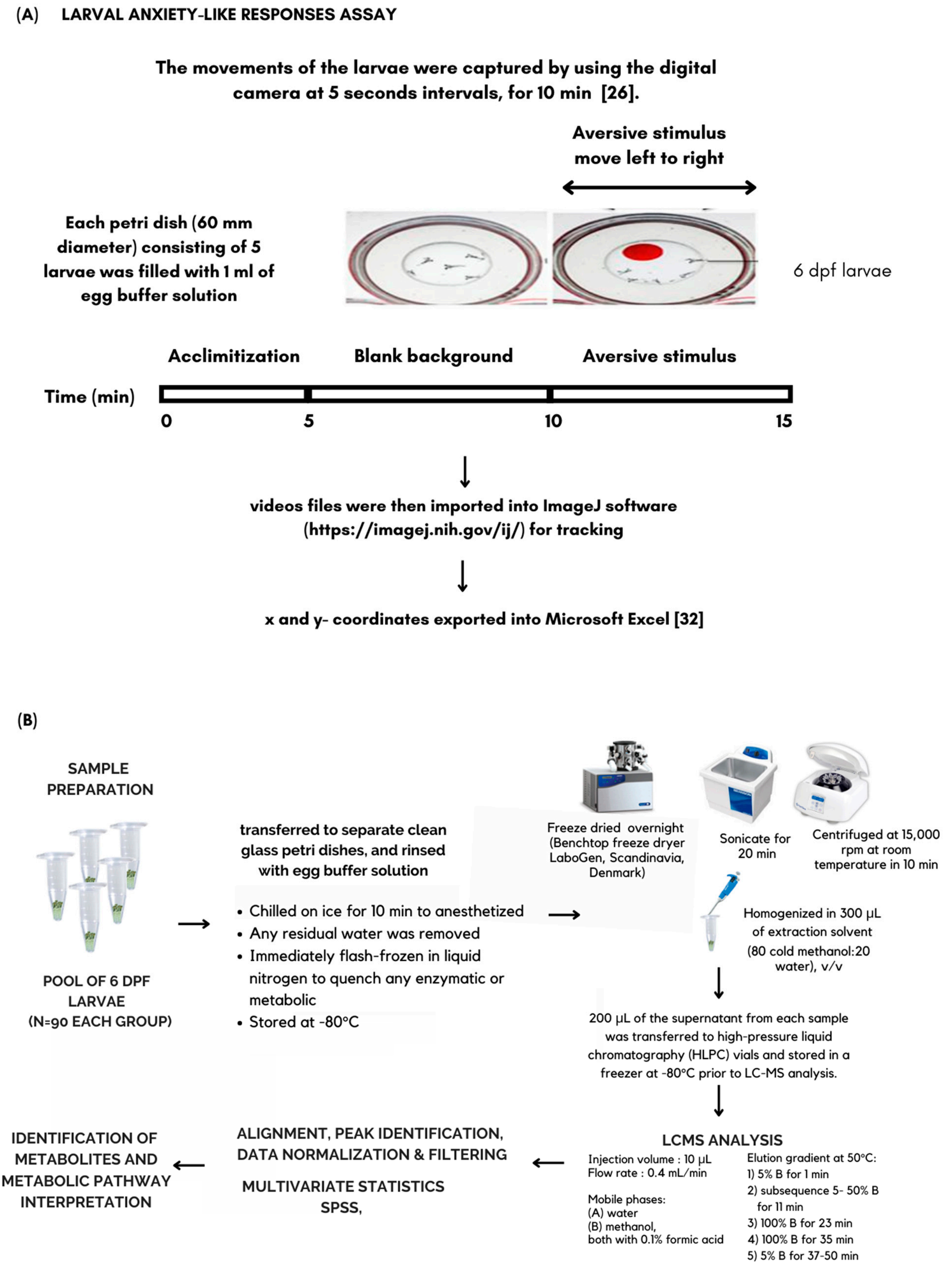

Figure S3. Summary of (**A**) anxiety-like response and (**B**) sample preparation for LCMS analysisin 6 dpf larvae.

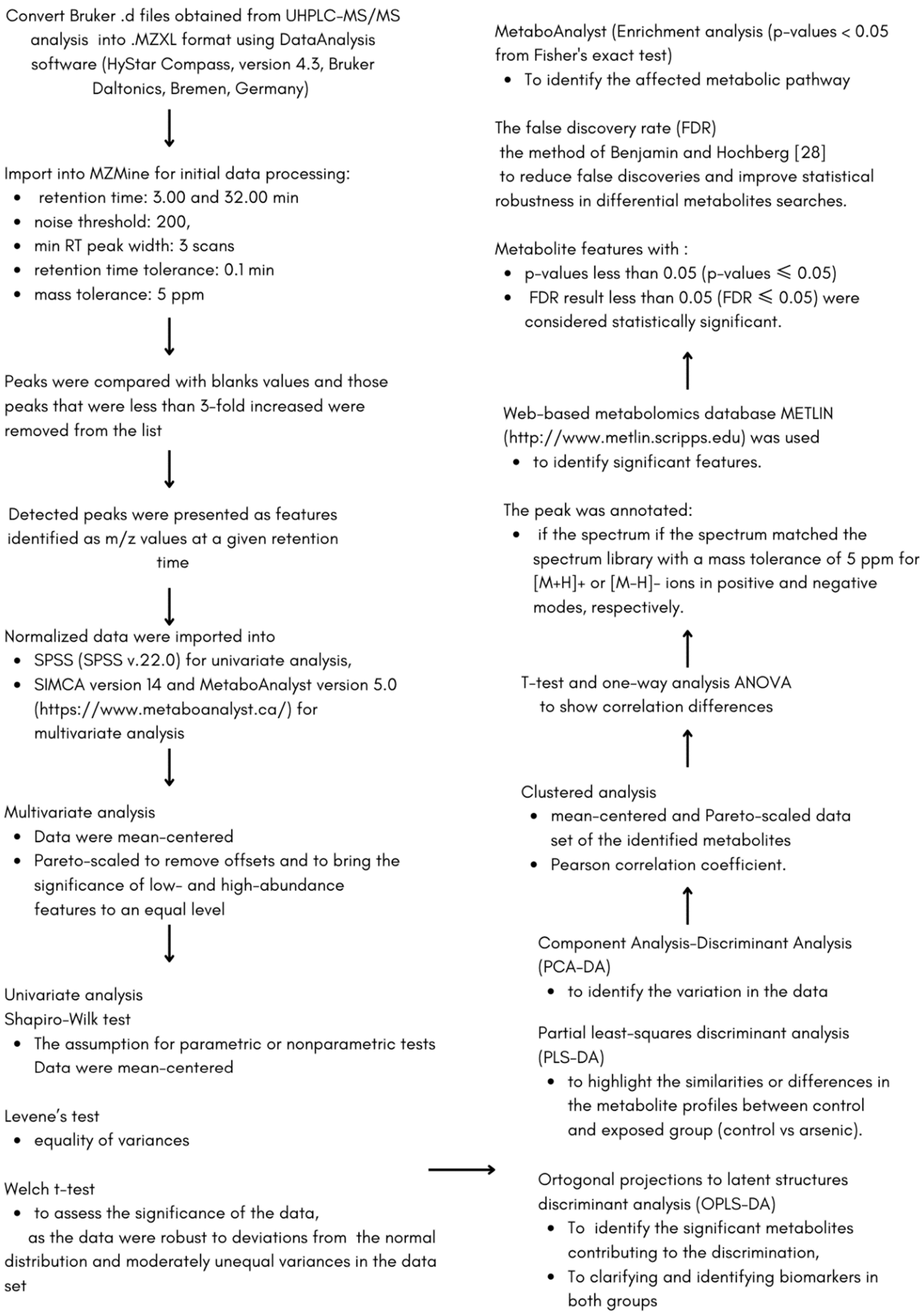

Figure S4. Summary of LCMS data analysis.

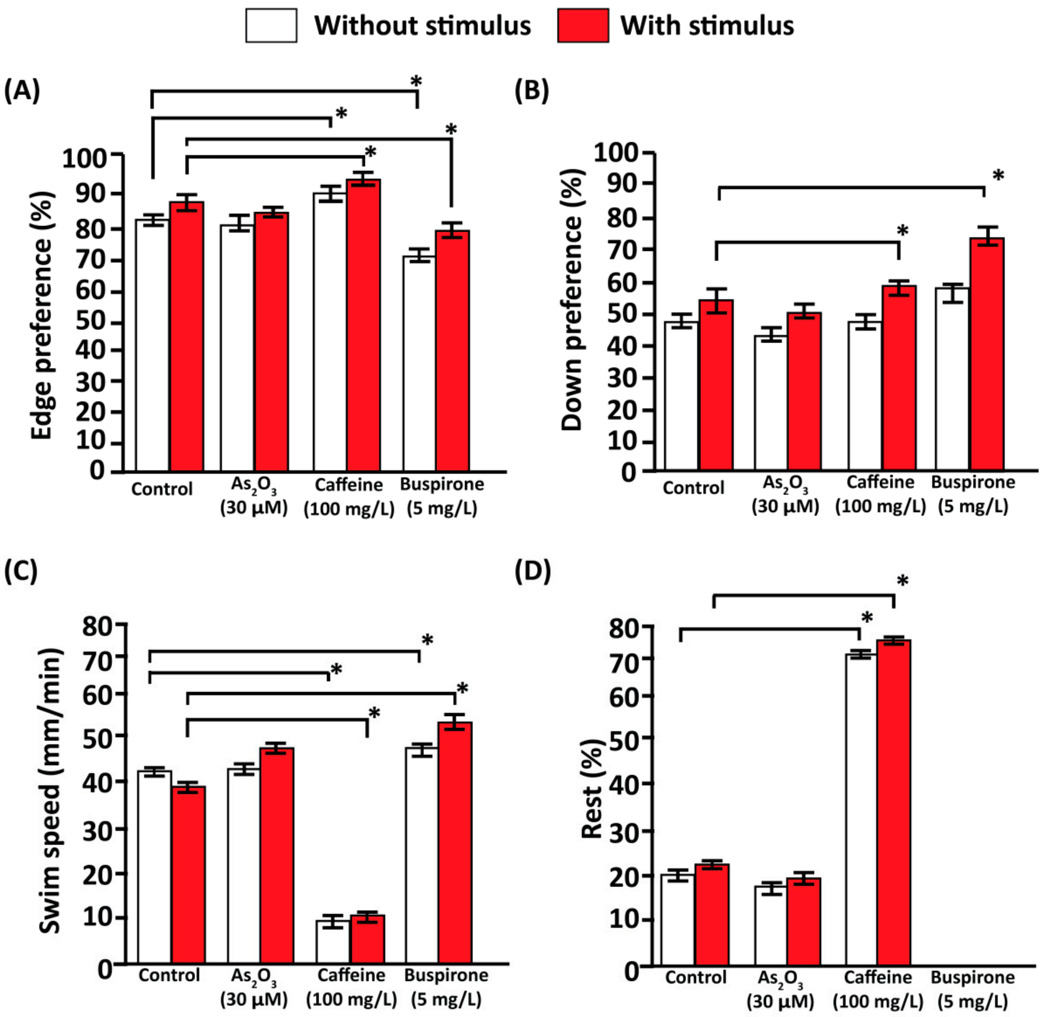

Figure S5. Effects of As_2O_3, caffeine and buspirone on anxiety-like responses of 6 dpf zebrafish larvae. (**A**–**D**) Larvae exposed to As_2O_3 caused no changes either on thigmotaxis (edge preference), avoidance (down preference), swimming speed or percentage of rest. (**A**–**D**) Exposure to caffeine caused increased in thigmotaxis, reduced avoidance, slower swimming speed and increased percentage of rest. (**A**–**D**) Larvae exposed to buspirone caused decreased in thigmotaxis, increased avoidance, elicit swimming speed and no resting larvae were detected. The white bar shows data from larvae exposed to a blank background and the red bars show the larvae exposed to red moving ball in the Microsoft PowerPoint as aversive stimulus. Data shown as mean ± SEM of triplicate wells (n = 90 embryos per exposure group), significantly different from control *($p \leq 0.05$).

(A)

(B)

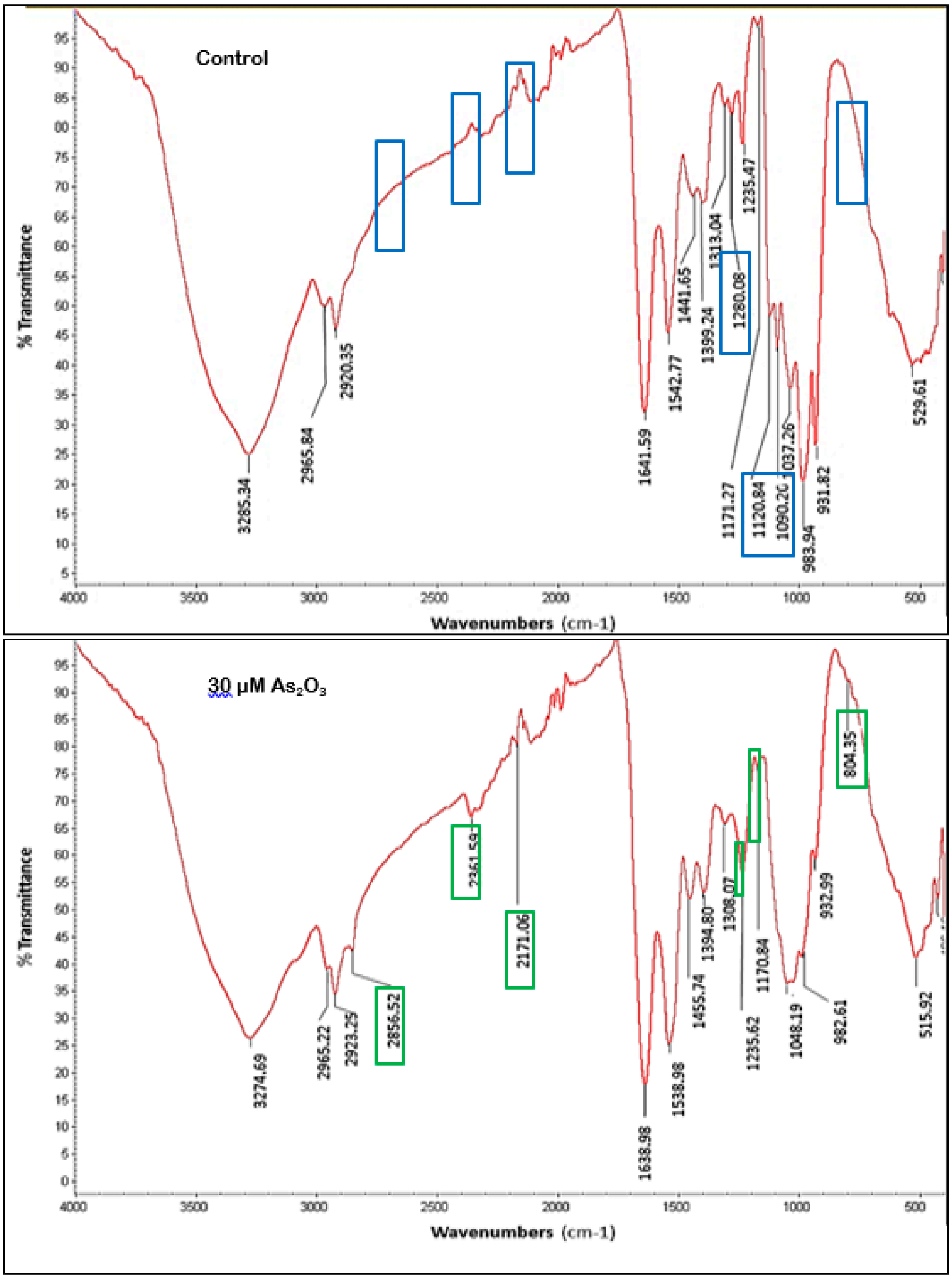

Figure S6. General band assignment of the FTIR spectra of (**A**) control and (**B**) 30 μM As_2O_3-exposed zebrafish larvae in the 500–4000 cm^{-1} regions. The rectangular shapes show the missing or additional peaks upon As_2O_3 exposure as compared with the control.

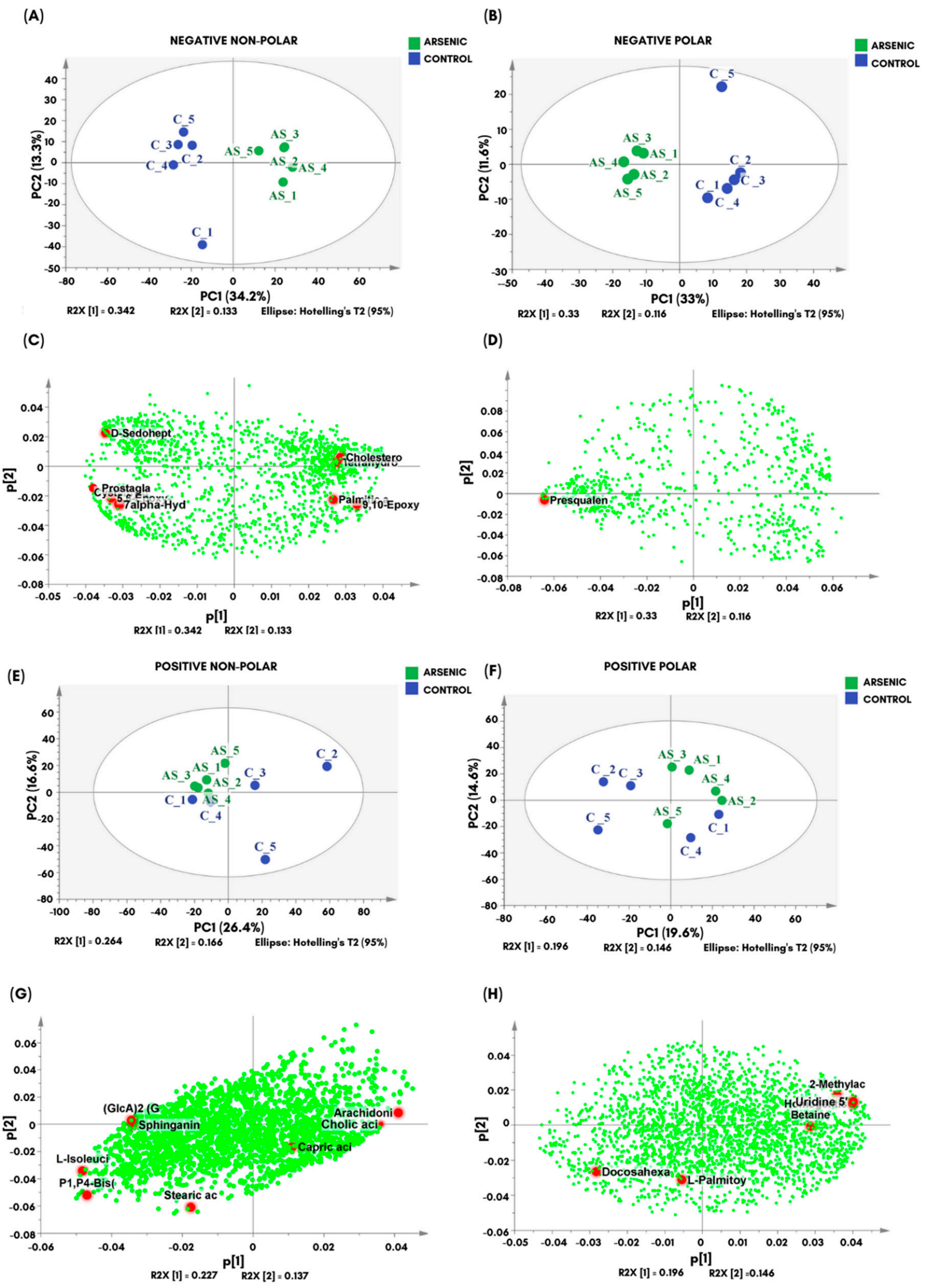

Figure S7. PCA score plot (**A**,**B**,**E**,**F**) and loading scatter plot (**C**,**D**,**G**,**H**) of negative and positive modes based on the normalized data of zebrafish larvae exposed to 30 μM As_2O_3 compared to control for non-polar and polar features.

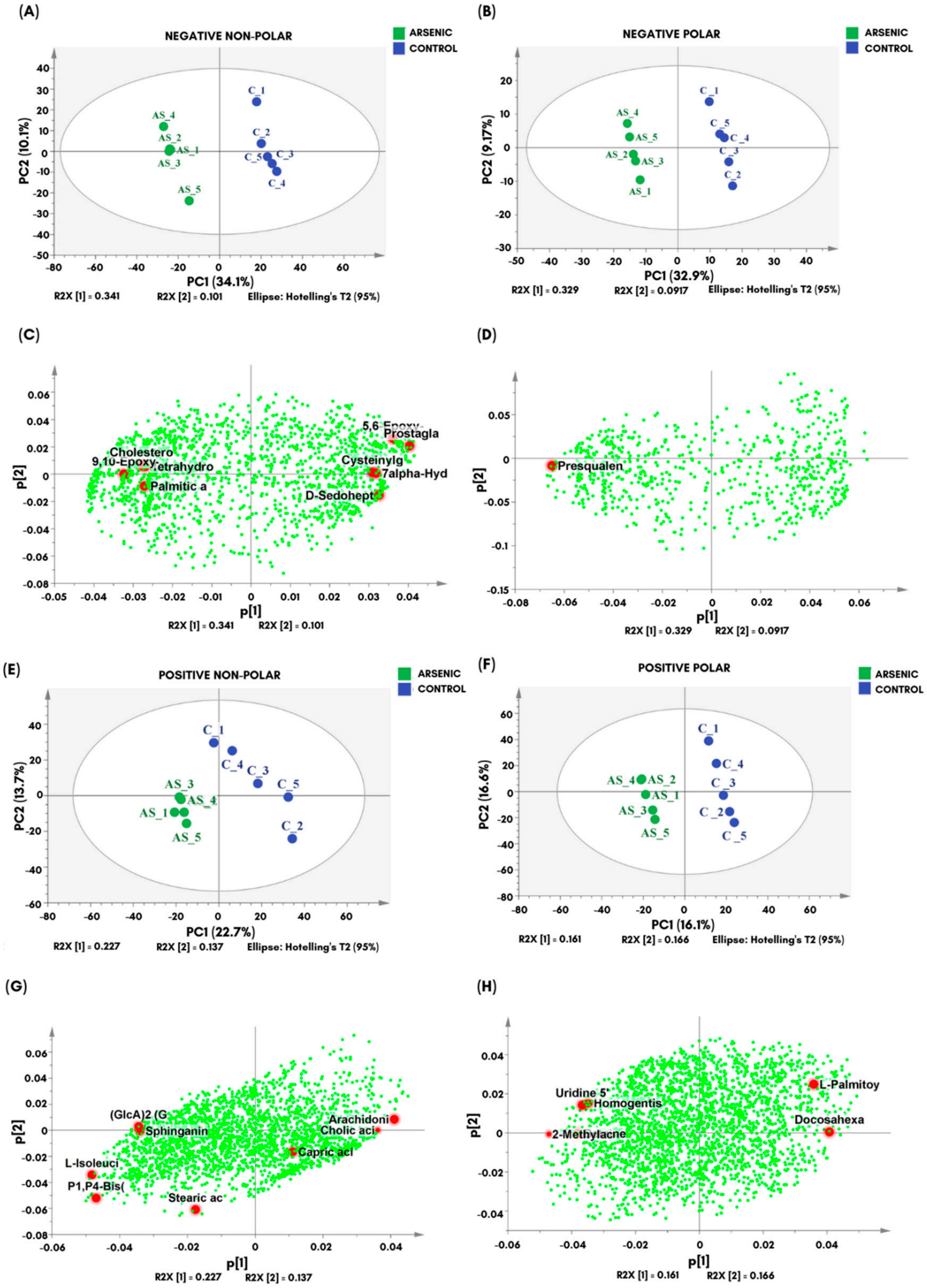

Figure S8. PLS–DA score plot (**A**–**D**) and loading scatter plot of negative modes based on the zebrafish larvae normalized data exposed to 30 μM As_2O_3 in comparison to control for non-polar and polar features. (**E**–**H**) PCA–DA score plot (**A**–**D**) and loading scatter plot of positive modes based on the normalized data for non-polar and polar features.

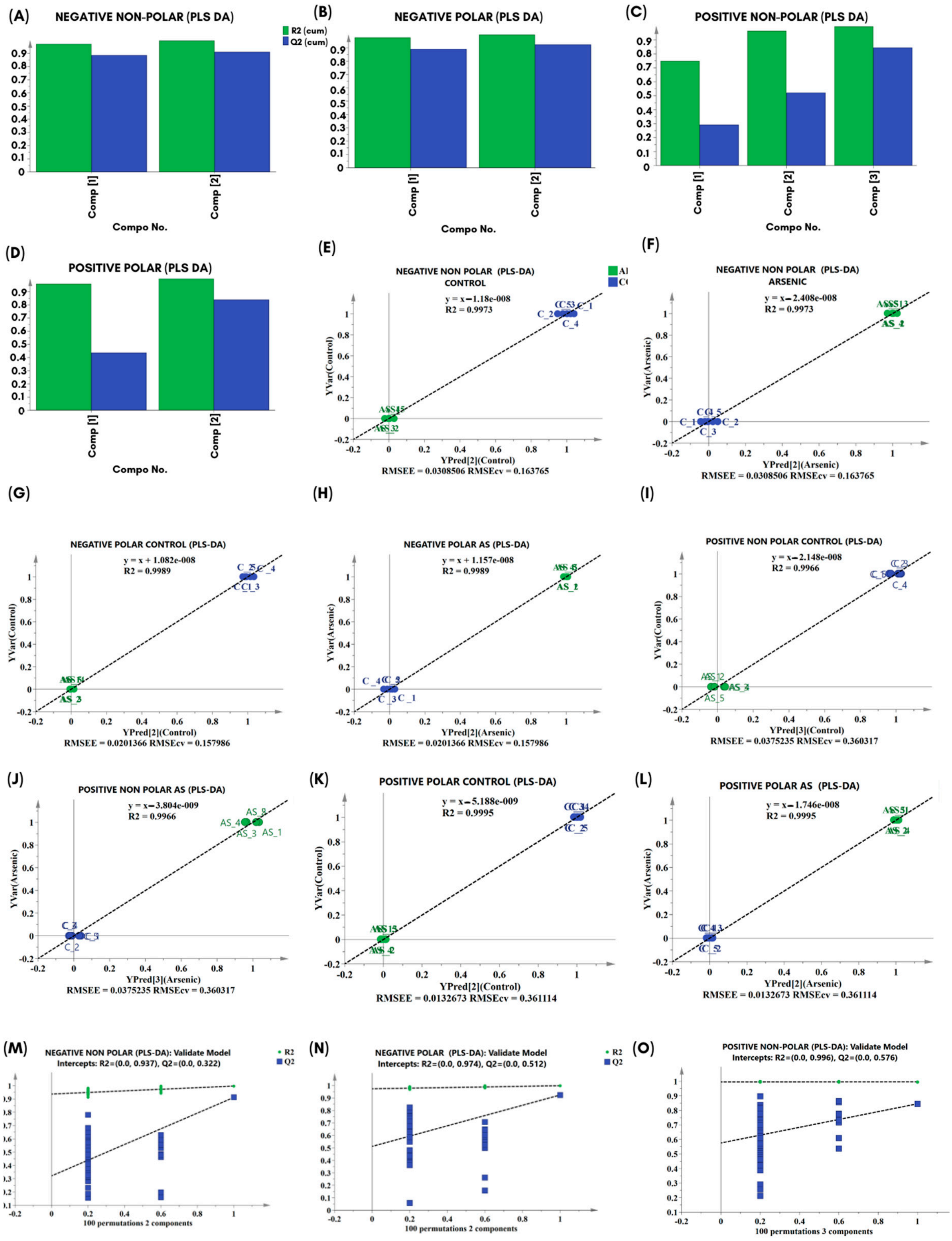

Figure S9. PLS–DA validating models of negative and positive mode based on the zebrafish larvae normalized data exposed to 30 μM As_2O_3 in comparison to control for non-polar and polar features. (**A–O**)

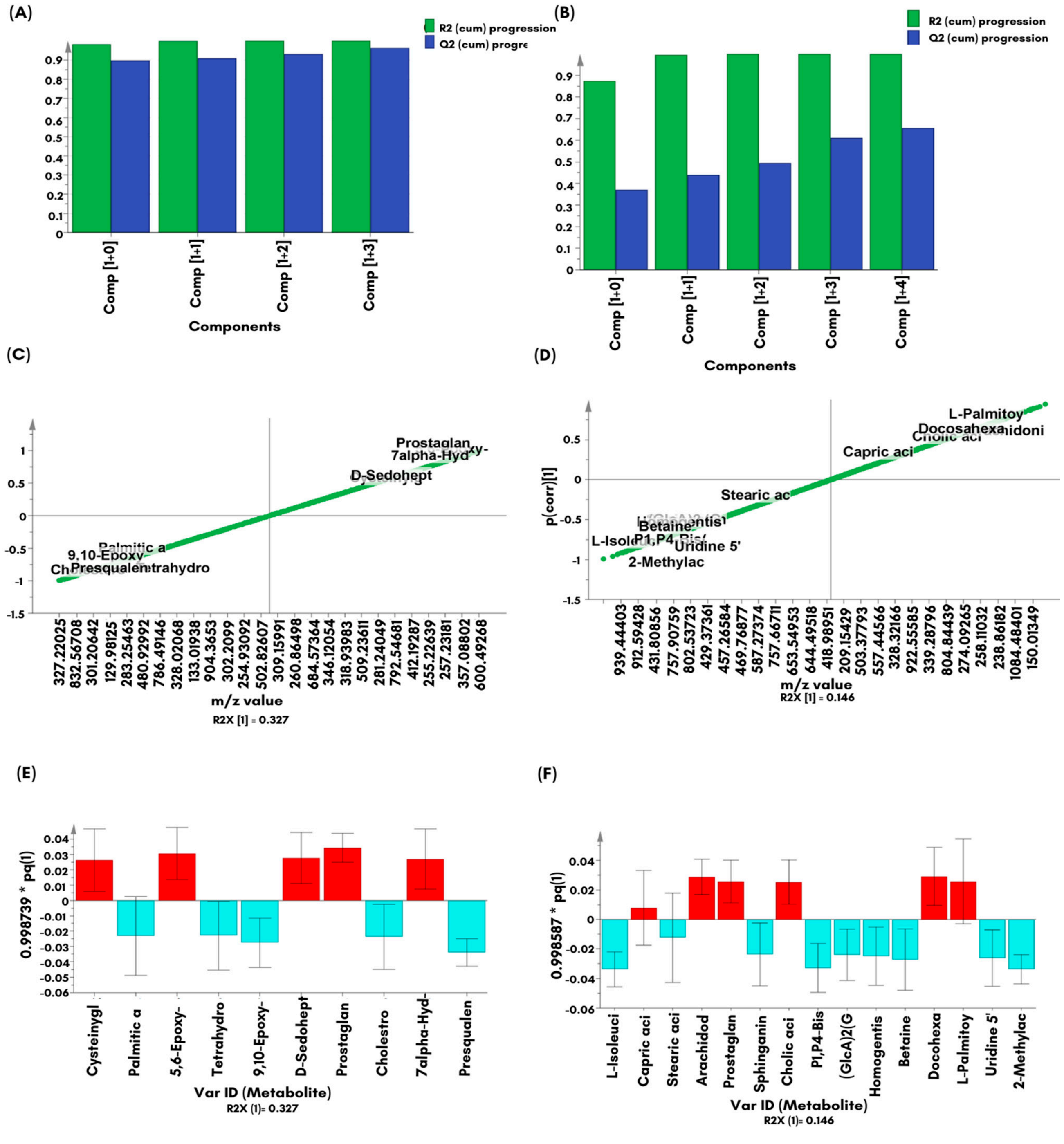

Figure S10. OPLS–DA Summary of fit of negative (**A**) and positive (**B**) modes based on the zebrafish larvae normalized data exposed to 30 μM As_2O_3 in comparison to control. S-plot negative (**C**) and positive (**D**) modes derived from the OPLS–DA model indicating biomarkers for control (upper right) and As_2O_3 (lower left) in zebrafish larvae. Loading column for negative (**E**) and positive (**F**) modes. Blue column represent the downregulated metabolites whereas red

column represent upregulated.

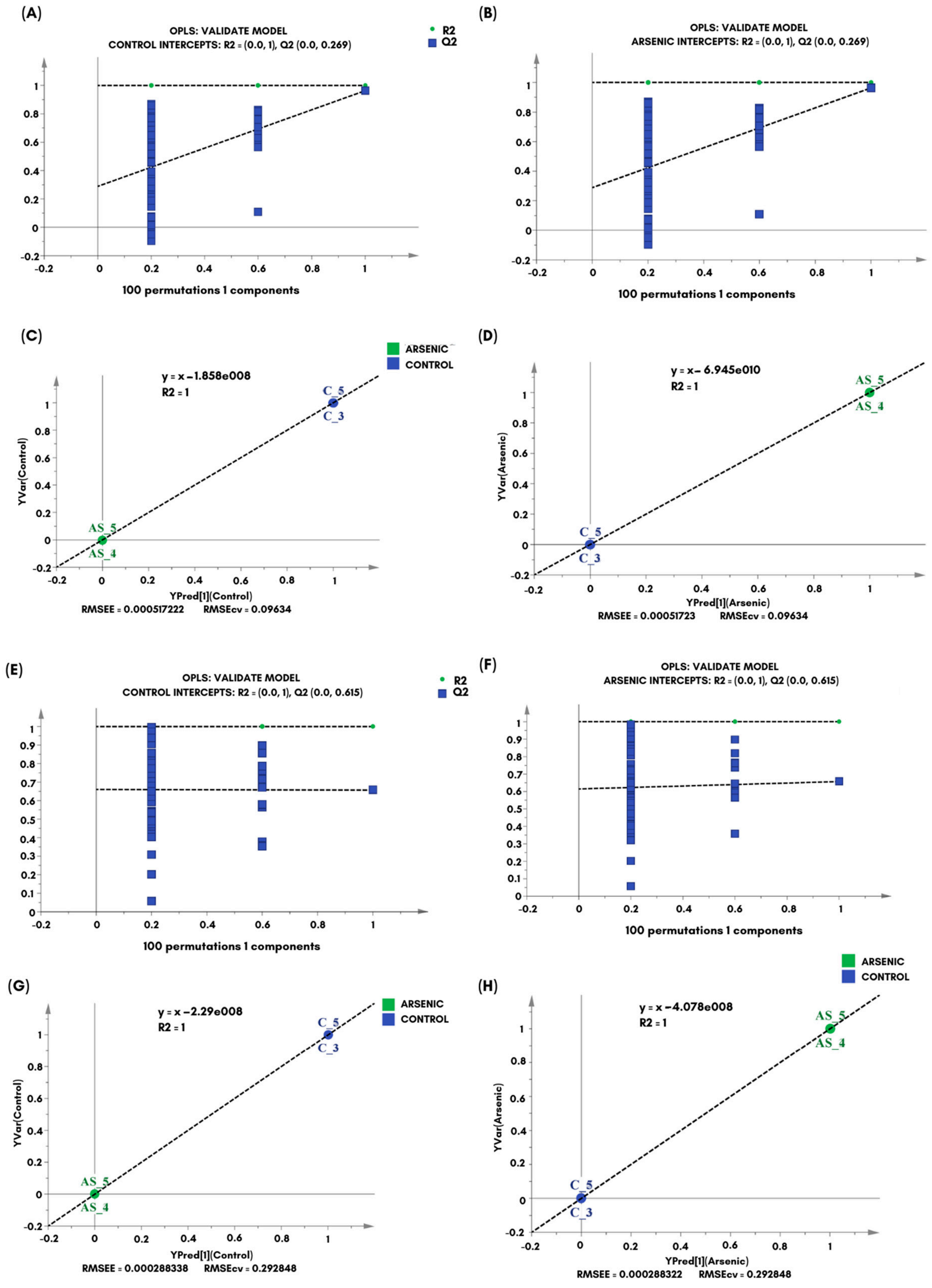

Figure S11. OPLS–DA validating models (**A**–**D**) of negative and (**E**–**H**) positive mode based on the zebrafish larvae normalized data exposed to 30 μM As_2O_3 in comparison to control.

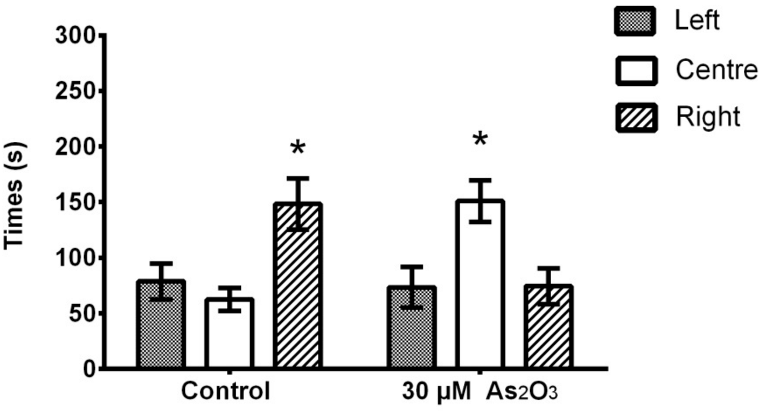

Figure S12. Control adult zebrafish had greater preference to right chamber compared to As_2O_3 exposed zebrafish that had lost directional preference.

Table S1. Designated forward and reverse sequence of selected genes associated with ASD based on available references.

Copyright: © 2022 by the authors. Licensee MDPI, Basel, Switzerland. This article is an open access article distributed under the terms and conditions of the Creative Commons Attribution (CC BY) license (https://creativecommons.org/licenses/by/4.0/).

Genes	Primers	Reference
adsl	F: CCAGCAAAGAGATGGCCTAC<br>R: TGACATCATGCCTGAGCTTC	NM_199899.2
Nrxn	F: GAGCAGTAGCGATGAGATTAC<br>R: ACTACCGCCGACATAGAA	NP_001073490.1
shank3a	F: GAGGTAGAGGAGGAGGATTT<br>R: CATGGGAGGATGTAGTTTACG	[48]
tsc1b	F: AGCTGCAACACCTCCTCCTA<br>R: TCACCTCTGCCATCTCTGTG	NM_001282392.1
β-actin	F: TCACCTCTCTTGCTCCTT<br>R: TAGGTTGGTCGTTCGTTTG	BC045846.1

Table S2. General band assignment of the FTIR spectra of control and 30 µM As_2O_3 exposed zebrafish larvae. The FTIR spectrum of 6 dpf zebrafish larvae showed a complex of several bands originating from functional groups belonging to lipids, proteins, nucleic acids, and carbohydrates (↓-decreased, ↑- increased).

Frequency (cm^{-1})		Bonds	Functional Groups	Components
**Control**	**30 µM As_2O_3**			
3285.34	3274.69 ↓	N-H stretch	Amide	Proteins

2965.84	2965.22 ↓	C-H of CH_3 asymmetric stretch	Alkanes	Lipids
2920.35	2923.25 ↑	C-H of CH_3 asymmetric stretch	Alkanes	Lipids
Not observed	2856.52	C-H of CH_2 asymmetric stretch	Alkanes	Lipids
Not observed	2361.59	C-H stretch	Alkenes	Lipids
Not observed	2171.06	C-N stretch	Nitrile	Lipids
1641.59	1638.43 ↓	C=O	Amide	Proteins
1542.77	1538.98 ↓	C–N stretching, N–H bending	Amide	Proteins
1441.65	1455.74 ↑	methyl C–H asymmetric bending	Alkanes	Lipids
1399.24	1394.80 ↓	COO− symmetric stretching	Carboxylic acids	Fatty acids, amino acids
1313.04	1308.07 ↓	C-O stretch	Alcohol, Carboxylic acids	Carbohydrate
1280.08	Not observed	PO_2^- asymmetric stretch	Phosphate	Nucleic acid, lipids (phospholipids)
1235.47	1235.62 ↑	PO_2^- asymmetric stretch	Phosphate	Nucleic acid, lipids (phospholipids)
1171.24	1170.84 ↓	C–O asymmetric stretching	Alcohol, Carboxylic acids	Nucleic acid, carbohydrates
1120.84	Not observed	C-N, C-O stretch	Amines, Alkyl halides, Carboxylic acids, Esters	Carbohydrates
1090.20	Not observed	PO_2^- symmetric stretch	Phosphate	Nucleic acid, lipids (phospholipids)
1037.26	1048.19 ↑	C-O stretch	Alcohol, Carboxylic acids	Carbohydrate
983.94	982.61 ↓	C-C, C-H bend	Alkenes	Nucleic acid
931.82	932.99 ↑	C–C,C-H stretch	Alkenes	Nucleic acid
Not observed	804.35	C-N stretch	Aromatic amines	Amino acid
529.61	515.92 ↓	S-S stretch	Polysulfides	Amino acids

Table S3. List of identified and significant metabolites in As_2O_3 exposed larvae in comparison to the control ($p < 0.05$).

Mode	m/z	RT (min)	Compound	As_2O_3 vs Control				Reference		
				P-value	Fold change	Log_2 Fold change	FDR	HMDB	PUBCHEM	KEGG
**Negative**	433.1423	3.53	Prostaglandin H_2	0.0000	55.4020	−5.7919	0.0003	HMDB0001381	445049	C00427
**Negative**	341.2099	7.69	5,6-Epoxy-8,11,14-eico-satrienoic acid	0.0013	4.6285	2.2106	0.0178	HMDB0002190	5283202	C14768
**Negative**	355.2497	7.48	9,10-Epoxyoctadecenoic acid	0.0084	0.3396	−1.5579	0.0543	HMDB0004701	6246154	C14825
**Negative**	467.2764	6.44	7α-Hydroxy-3-oxo-4-cholestenoate	0.0106	22.5310	4.4938	0.0624	HMDB0012458	3081085	C17337
**Negative**	466.3075	7.31	Cholesterol sulfate	0.0346	0.0333	−4.9070	0.1299	HMDB0000653	65076	C18043
**Negative**	257.2310	7.06	Palmitic acid	0.0409	0.2051	−2.2859	0.1404	HMDB0000220	985	C00249
**Negative**	351.2369	7.89	Tetrahydrocorticosterone	0.0431	0.0069	−7.1738	0.1404	HMDB0000268	9863245	C05476
**Positive**	267.2689	7.59	Stearic acid	0.0043	2.8937	1.5329	0.3206	HMDB0000827	5281	C01530
**Positive**	351.2299	7.10	Docosahexaenoic acid	0.0083	0.2194	−2.1881	0.2891	HMDB0002183	445580	C06429
**Positive**	269.2269	9.88	Arachidonic acid	0.0097	0.2796	−1.8384	0.4002	HMDB0001043	444899	C00219
**Positive**	400.3432	7.36	L-Palmitoyl-carnitine	0.0296	0.2634	−1.9245	0.4714	HMDB0000222	11953816	C02990

**Positive**	97.02820	1.47	Homogentisic acid	0.0358	2.2277	1.1556	0.4998	HMDB0000130	780	C00544
**Positive**	338.2822	8.78	Sphinganine 1-phosphate	0.0491	2.3767	1.249	0.5810	HMDB0001383	644260	C01120
